# Defining critical roles for NF-κB p65 and type I interferon in innate immunity to rhinovirus

**DOI:** 10.1002/emmm.201201650

**Published:** 2012-11-14

**Authors:** Nathan W Bartlett, Louise Slater, Nicholas Glanville, Jennifer J Haas, Gaetano Caramori, Paolo Casolari, Deborah L Clarke, Simon D Message, Julia Aniscenko, Tatiana Kebadze, Jie Zhu, Patrick Mallia, Joseph P Mizgerd, Maria Belvisi, Alberto Papi, Sergei V Kotenko, Sebastian L Johnston, Michael R Edwards

**Affiliations:** 1 Department of Respiratory Medicine, National Heart Lung Institute, Imperial College LondonLondon, UK; 2 MRC and Asthma UK Centre in Allergic Mechanisms of AsthmaLondon, UK; 3 Centre for Respiratory Infections, Imperial College LondonLondon, UK; 4 Sezione di Malattie dell'Apparato Respiratorio, Centro per lo Studio delle Malattie Infiammatorie Croniche delle Vie Aeree e Patologie Fumo Correlate dell'Apparato Respiratorio (CEMICEF), University of FerraraFerrara, Italy; 5 Respiratory Pharmacology, National Heart and Lung Institute, Imperial College LondonLondon, UK; 6 Imperial College Healthcare National Health Service TrustLondon, UK; 7 The Pulmonary Centre, Boston University School of MedicineBoston, Massachusetts, USA; 8 Department of Biochemistry and Molecular Biology, University Hospital Cancer Center, UMDNJ-New Jersey Medical SchoolNewark, NJ, USA

**Keywords:** asthma, inflammation, interferon, NF-kappaB, rhinovirus

## Abstract

The importance of NF-κB activation and deficient anti-viral interferon induction in the pathogenesis of rhinovirus-induced asthma exacerbations is poorly understood. We provide the first *in vivo* evidence in man and mouse that rhinovirus infection enhanced bronchial epithelial cell NF-κB p65 nuclear expression, NF-κB p65 DNA binding in lung tissue and NF-κB-regulated airway inflammation. *In vitro* inhibition of NF-κB reduced rhinovirus-induced pro-inflammatory cytokines but did not affect type I/III interferon induction. Rhinovirus-infected *p65*-deficient mice exhibited reduced neutrophilic inflammation, yet interferon induction, antiviral responses and virus loads were unaffected, indicating that NF-κB p65 is required for pro-inflammatory responses, but redundant in interferon induction by rhinoviruses *in vivo*. Conversely, *IFNAR1*^−/−^ mice exhibited enhanced neutrophilic inflammation with impaired antiviral immunity and increased rhinovirus replication, demonstrating that interferon signalling was critical to antiviral immunity. We thus provide new mechanistic insights into rhinovirus infection and demonstrate the therapeutic potential of targeting NF-κB p65 (to suppress inflammation but preserve anti-viral immunity) and type I IFN signalling (to enhance deficient anti-viral immunity) to treat rhinovirus-induced exacerbations of airway diseases.

→ See accompanying article http://dx.doi.org/10.1002/emmm.201202032

## INTRODUCTION

The immune response to virus infections involves a robust innate anti-viral response meditated by type I and III interferons (IFNs) and a potent inflammatory response involving both rapid immune cell recruitment and damage of infected cells and tissues. The regulation of type I IFN-β, IFN-αs and type III IFN-λs has been well studied with IFN-β being used as a model system of eukaryotic gene expression since the early 1990s (Du et al, 1993). The IFN-β promoter contains four positive regulatory domains (PRDs), which allow the DNA binding of distinct transcription factors (Du et al, 1993; Falvo et al, 2000; Thanos & Maniatis, 1995; Wathelet et al, 1998). These transcription factors include IRF-1, IRF-3 and IRF-7 as well as the heterodimeric ATF/c-Jun and members of the NF-κB or Rel family, which is composed of five related proteins, p65 (Rel A, NF-κB3), p50 (NF-κB1, precursor of which is p105), p52 (NF-κB2, precursor of which is p100), c-Rel and Rel B. Rel proteins have a central role in innate immunity with NF-κB p65 implicated in expression of type I and type III IFNs and pro-inflammatory cytokines.

Numerous studies over the years including electromobility shift assays (Thanos & Maniatis, 1995), X-ray crystallography (Berkowitz et al, 2002; Panne et al, 2007) and nucleosome analysis of the IFN-β locus (Apostolou & Thanos, 2008) have overwhelmingly supported the current paradigm that all of these transcription factors are required for virus-induced IFN-β transcription; although subtle differences in different cell types have been reported. The recent use of cells from gene-deficient mice (Wang et al, 2007) have questioned the role of NF-κB p65 for IFN-β gene expression, with two recent studies from the same laboratory providing conflicting data (Wang et al, 2007, 2010). A recent editorial on this subject (Balachandran & Beg, 2011) proposes that NF-κB family members including p65 are required to maintain basal activation of the IFN-β promoter or are required very early during infection before IRF-3 activation is optimal. Furthermore, all studies to date report that p65 is also required for IFN-λ gene expression, (Onoguchi et al, 2007; Osterlund et al, 2007; Siegel et al, 2011). Importantly, the current data for IFN-β and IFN-λ gene expression is entirely based on *in vitro* studies mostly utilizing cell lines or gene-deficient murine embryonic fibroblasts (MEFs) with model viruses that are not important human pathogens. The role of NF-κB p65 in IFN-β and IFN-λ production has never been investigated *in vivo* and the wider implications of the selective targeting of NF-κB p65 in important human diseases caused by virus infections is a subject of much interest yet one poorly addressed in mouse models of human disease.

Considering the lack of studies investigating the importance of p65 in IFN induction by important human viruses *in vivo*, we have investigated the role of NF-κB p65 and type I IFN signalling in the host defence and inflammatory response to human rhinovirus (RV) *in vivo* and *in vitro*. RVs are responsible for a range of severe human illnesses including acute exacerbations of lower airways diseases such as asthma (Johnston et al, 1995, 2005). A cardinal feature of RV infection *in vitro* is production of pro-inflammatory molecules, the expression of which is transcriptionally regulated by members of the NF-κB transcription factor family (Zhu et al, 1996, 1997). In asthma exacerbations, increased airway inflammation is strongly associated with clinical illness severity (Message et al, 2008; Papi et al, 2006; Wark et al, 2002) and this is thought to be mediated by NF-κB p65 activation, although studies directly demonstrating activated NF-κB p65 during RV infection or RV-induced asthma exacerbations *in vivo* are yet to be reported.

Asthmatic subjects experience significantly increased lower respiratory tract symptoms following either natural (Corne et al, 2002) or experimental RV infection (Message et al, 2008). Impaired antiviral immunity is likely to explain this increased susceptibility to RV infection as deficient type I and type IIII IFN production by asthmatic bronchial epithelial cells (Contoli et al, 2006; Uller et al, 2010; Wark et al, 2005) and bronchoalveolar lavage (BAL) macrophages (Contoli et al, 2006) has been observed *ex vivo*, with the latter related to increased virus load and exacerbation severity *in vivo*. These relationships suggest, but are unable to definitively show, a causal relationship between IFN deficiency and disease severity. These data, along with the data reporting a requirement of p65 for IFN gene expression, suggest that approaches that inhibit p65 would suppress RV-induced IFN in the asthmatic lung, further impairing antiviral immunity, increasing virus loads, virus-induced inflammation and exacerbation severity. Therefore, understanding whether or not NF-κB p65 is involved in IFN production to RV is extremely important and this key issue is yet to be addressed as there are no data reported on the causal role of p65 or type I IFN in lung host defence against RV infection using *in vivo* human and mouse models of RV. Further, the requirement of p65 for RV-induced antiviral IFN expression in human bronchial epithelial cells (HBECs) *in vitro* is also unknown. Determining the contribution of p65 to IFN-mediated antiviral and pro-inflammatory responses is vital to identifying therapeutic targets for RV-induced lower airway diseases. Demonstrating that IFN is critical for antiviral responses to RV *in vivo* is necessary to justify further development of IFN-based therapies (Hayden & Gwaltney, 1984; Koltsida et al, 2011).

This is the first report investigating the role of p65 in immunity to a virus *in vivo* and includes combined studies in human and mouse models of RV infection to demonstrate that NF-κB p65 is a central regulator of RV-induced inflammation in the airways. Furthermore, we provide evidence that suppressing p65 expression, whilst reducing airways inflammation, did not affect IFN production or antiviral immune responses, despite over 20 years of *in vitro* experiments that suggest the contrary. Inhibition of p65 is therefore identified as an attractive target for development of anti-inflammatory therapies that would not further impair IFN responses in virus-induced asthma exacerbations. In doing so, we have also provided clear evidence that responses mediated by type I IFN *in vivo* are critical for antiviral responses to RV thereby identifying IFN as another therapeutic approach likely to be beneficial.

## RESULTS

### NF-κB is activated by RV infection *in vivo* in the lung and *in vitro* in primary bronchial epithelial cells

**Figure 1:**
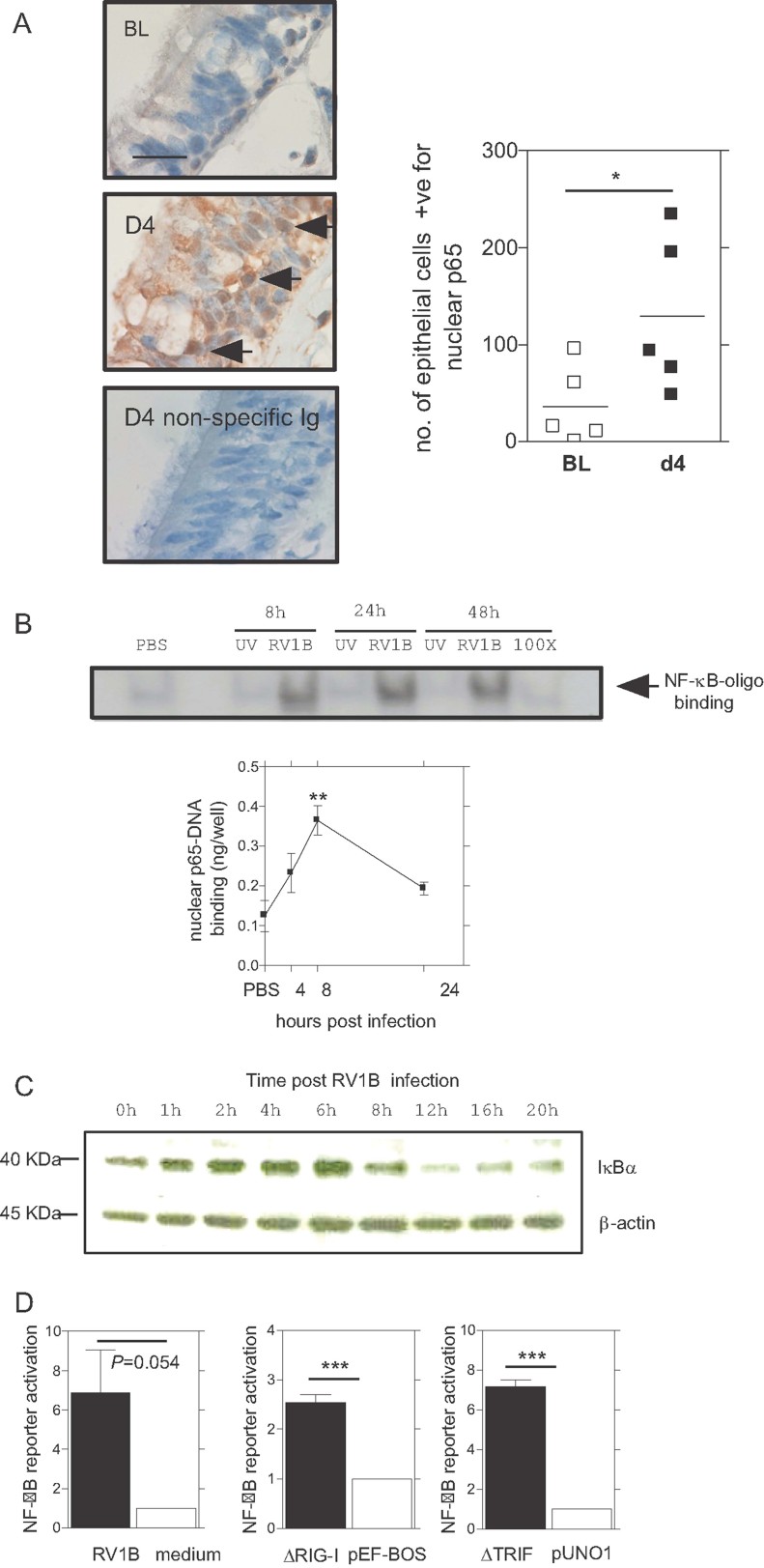
RV and RV-mediated signalling pathways activate NF-κB *in vitro* and *in vivo* Healthy human subjects (*n* = 5) were infected with RV16 and bronchial biopsies were taken prior to (BL) and D4 after infection and stained to quantify bronchial epithelial cell nuclear NF-κB p65. Horizontal line indicates a scale of 20 µM. Arrows indicate nuclear p65 staining. The negative control was stained with non-specific rabbit Ig rather than primary rabbit anti-p65 antibody. Graph shows each data point with mean, differences between groups were identified by *t*-test *p < 0.05.BALB/c mice were infected intranasally with RV1B. Negative controls were dosed with UV-inactivated RV1B or PBS intranasally and NF-κB activation in lung nuclear protein extract assessed by NF-κB-DNA binding in EMSA (*upper panel*). Presence of p65 in RV infection *in vivo* was confirmed by measuring nuclear p65-DNA binding in a timecourse performed in BL/6 129 mice (*lower panel*). Data was analysed by one-way ANOVA (*n* = 1 experiment, five mice per group) **p < 0.01.HBECs were infected with RV1B and IκBα degradation measured by Western blot.HBECs were infected with RV1B which caused activation of a minimal NF-κB promoter at 24 h compared to medium treated cells (*n* = 5 independent experiments). HBECs were transfected with plasmids encoding constitutively active molecules involved in RV sensing pathways, ΔRIG-I and ΔTRIF. Both ΔRIG-I and ΔTRIF induced NF-κB reporter activation relative to empty vector controls pEF-BOS and pUNO1, respectively (*n* = 6 independent experiments). Reporter data is presented as fold induction *versus* control and analysed by *t*-test ***p < 0.001 unless otherwise stated, as indicated all data are expressed as mean ± SEM.

As it is not known whether RV infection leads to activation of NF-κB p65 *in vivo*, we initially investigated this in human and mouse models of RV infection. Fig 1A shows increased activation of NF-κB p65 as assessed by p65 nuclear stained bronchial epithelial cells in bronchial biopsies, from baseline (BL) to day4 (D4) following experimental human RV infection (Message et al, 2008). NF-κB in lung tissue was also activated in a mouse model of RV infection. Induction of binding to labelled NF-κB-containing oligonucleotides was observed in nuclear protein extracted from whole lung of RV-infected mice. No signal was observed for mice dosed with UV-inactivated virus indicating that NF-κB activation was replication-dependent. NF-κB binding was also effectively competed with 100× excess unlabelled probe demonstrating NF-κB binding specificity (Fig 1B). Activation of p65 was confirmed by nuclear p65-DNA binding experiments performed over time in Bl/6 129 mice (Fig 1B). In HBECs, RV1B caused IκBα degradation from 8 h post-infection (Fig 1C) and NF-κB-dependent reporter gene activation (Fig 1D). Transfection of HBECs with plasmids expressing constitutively active forms of the RV RNA-sensing molecules RIG-I (ΔRIG-I) (Yoneyama et al, 2004) and TRIF (ΔTRIF; Slater et al, 2010; Yamamoto et al, 2003) also activated the NF-κB-dependent reporter (Fig 1D).

### Allergen challenge with RV infection increases NF-κB p65 activation and NF-κB-regulated cytokines and chemokines

**Figure 2:**
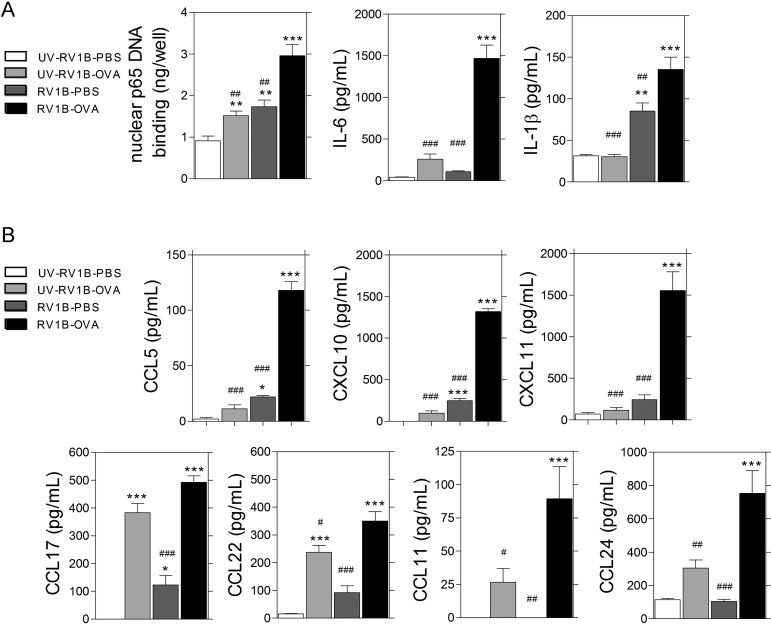
Exacerbation of allergic airway inflammation involves enhanced NF-κB p65 activation and NF-κB p65-responsive genes Balb/c mice were sensitized by i.p injection of OVA and challenged 10 days later on 3 consecutive days with either intranasal OVA or PBS administration; receiving intranasal RV1B or UV-RV1B on the third day. BAL and whole lung was harvested at 8 and 24 h. From whole lung, nuclear NF-κB p65 DNA binding was measured at 8 h post infection. RV1B-OVA, RV1B-PBS and UV-RV1B-OVA had significantly elevated p65 binding *versus* UV-RV1B-PBS, and RV1B-OVA treated mice had increased p65 binding *versus* the other groups. At 8 h post infection, RV1B-OVA mice had increased BAL pro-inflammatory cytokines IL-6, and IL-1β compared to RV1B-PBS or UV-RV1B-OVA treated groups.At 24 h post infection, RV1B-OVA mice had increased BAL CCL5, CXCL10, CXCL11, CCL17, CCL22, CCL11 and CCL24 compared to RV1B-PBS or UV-RV1B-OVA treated groups (*n* = 1 experiment with five mice per group or per time point). All data was analysed by one way ANOVA *p < 0.05, **p < 0.01, ***p < 0.001 *versus* UV-RV1B-PBS, ^#^*p* < 0.05, ^##^*p* < 0.01, ^###^*p* < 0.001 *versus* RV1B-OVA group. All data are expressed as mean ± SEM.

We have previously reported that RV infection exacerbates allergic airways inflammation (Bartlett et al, 2008). Using this model, we examined NF-κB p65 activation at early time points (8 h) observing that live RV infection during allergen challenge (RV1B-OVA) increased NF-κB p65 DNA binding in lung nuclear protein extracts when compared to live infection alone (RV1B-PBS) and allergen challenge together with inactivated RV (UV-RV1B-OVA; Fig 2A). Either RV infection alone (RV-PBS) or OVA challenge with inactivated RV1B (UV-OVA) induced more NF-κB p65 DNA binding than that observed in double-negative controls (UV-RV1B-PBS; Fig 2A). NF-κB p65 activation in the context of allergen and virus challenge was associated with markedly increased induction of IL-6, which was not induced by either stimulus alone, and clear further augmentation of IL-1β, which was induced by virus but not allergen alone (Fig 2A). The pleotropic chemokine CCL5, which is a chemoattractant for both granulocytes and lymphocytes, was highly synergistically increased in RV1B-OVA compared to all other groups (Fig 2B). Lymphocyte attracting chemokines CXCL10, CXCL11 CCL17 and CCL22 were also highest in RV1B-OVA as were the eosinophil-attracting chemokines CCL11 and CCL24 (Fig 2B). This data is direct *in vivo* evidence that RV infection in the allergic lung causes increased NF-κB p65 activation and expression of NF-κB-regulated pro-inflammatory cytokines and chemokines implicating this transcription factor in the pathogenesis of RV-induced exacerbation of allergic airway inflammation.

### Specific inhibition of NF-κB p65 does not suppress RV-induced IFNs but inhibited pro-inflammatory chemokine production in bronchial epithelial cells

**Figure 3:**
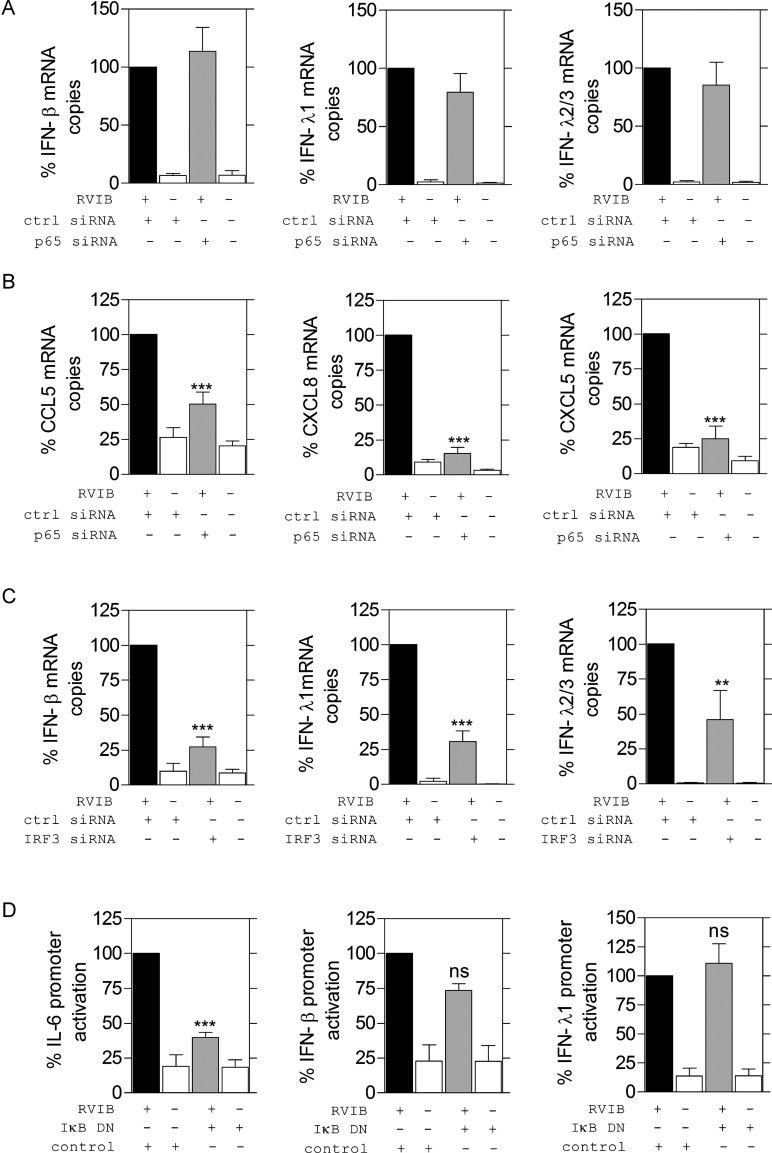
NF-κB p65 is not required for RV-induced IFN but for RV-induced pro-inflammatory cytokine expression in HBECs HBECs were transfected with siRNA specific to NF-κB p65 or irrelevant control siRNA and infected with RV1B or treated with medium (*n* = 5 independent experiments). IFN mRNA was assessed and expressed as % of mRNA copies relative to RV infected and transfected with control siRNA. The p65 specific siRNA did not significantly reduce RV1B induced IFN-β, IFN-λ1 or IFN-λ2/3 at 24 h post infection.HBECs were transfected with siRNA specific to NF-κB p65 or irrelevant control siRNA and infected with RV1B or treated with medium (*n* = 5). Pro-inflammatory cytokine mRNA was assessed and expressed as % of mRNA copies relative to RV infected and transfected with control siRNA. The p65 specific siRNA did significantly reduce RV1B induced CCL5, CXCL8 and CXCL5.HBECs were transfected with siRNA specific to IRF3 or irrelevant control siRNA and infected with RV1B or treated with medium (*n* = 5 independent experiments). IFN mRNA was assessed and expressed as % of mRNA copies relative to RV infected and transfected with control siRNA. IRF3 specific siRNA reduced IFN-β, IFN-λ1 and IFN-λ2/3 mRNA at 24 h.BEAS-2B cells were transfected with a plasmid encoding IκBα DN or empty vector control along with a *Renilla* luciferase plasmid and the IL-6, IFN-β or IFN-λ1 promoters and infected with RV1B for 24 h. Lysates were harvested and firefly and *Renilla* luciferase measured. Reporter data is presented as a ratio of firefly/*Renilla* and converted to a % of RV1B infected empty vector control (*n* = 4 independent experiments). All data was analysed by one way ANOVA **p < 0.01 and ***p < 0.001 *versus* control siRNA or control vector plus RV1B, data are expressed as mean ± SEM.

The major IFN subtypes induced by RV infection of bronchial epithelial cells are IFN-β and IFN-λ (Khaitov et al, 2009). Using small interfering RNA (siRNA) in HBECs, we showed that NF-κB p65 was not required for RV-induced IFN-β, IFN-λ1 or IFN-λ2/3 at 24 h (Fig 3A). In the same experiments, p65-specific siRNA significantly and almost completely prevented RV-induced expression of pro-inflammatory chemokines CCL5, CXCL8 and CXCL5 (Fig 3B). In contrast to p65, IRF3 was required for RV-induced IFN-β and IFN-λ1 and IFN-λ2/3 mRNA expression (Fig 3C). Specific siRNAs reduced both target mRNA and protein levels (Supporting Information Figs S1 and S2) and, in the absence of RV, did not induce IFN or chemokine expression (Supporting Information Tables SI and SII) confirming the role of these transcription factors in expression of RV-induced genes. Furthermore, p65 siRNA did not affect expression of other Rel family members (Supporting Information Tables SIII) and IRF3 siRNA did not inhibit expression of IRF1, IRF5, IRF7 and IRF9 mRNA (Supporting Information Tables SIV). To further confirm that NF-κB p65 was not required for RV-stimulated IFN-β or IFN-λ expression, we investigated stimulation of wild-type IFN promoters by RV in the presence or absence of an IκBα dominant-negative mutant (DN). IκBα is the major IκB species that prevents p65 nuclear translocation (Urban & Baeuerle, 1990; Zabel & Baeuerle, 1990). We found that RV-induced IL-6 expression was significantly reduced by the IκBα DN, however, RV could still drive IFN-β and IFN-λ1 promoter activation in the presence of the IκBα DN, providing further evidence that NF-κB p65 activation is not essential for RV-induced IFN expression (Fig 3D).

### Unlike NF-κB p65, type I IFN signalling is critical for antiviral responses to RV

**Figure 4:**
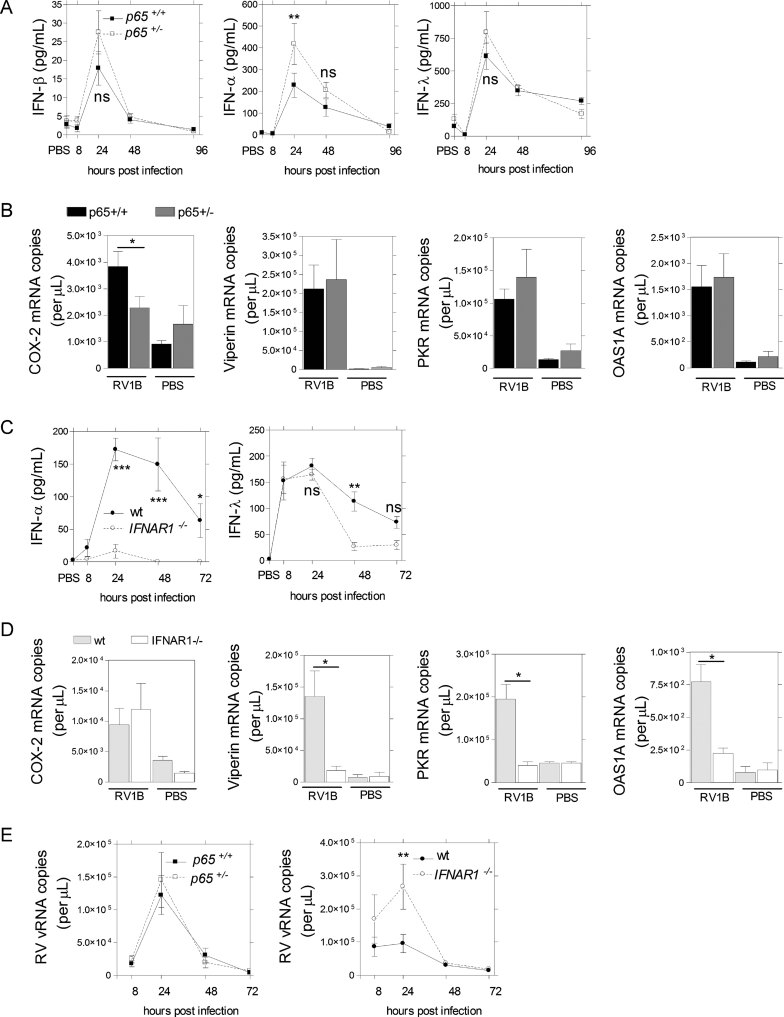
*P65^+/−^* mice exhibit robust antiviral responses Mice were inoculated with intranasal RV1B or PBS and harvested at various time points. NF-κB *p65*^+/+^ and *p65*^+/−^ mice had similar BAL IFN-β levels at 8–96 h post infection, while *p65*^+/−^ mice had significantly increased BAL IFN-α at 24 h but similar BAL IFN-λ levels at 8–96 h post infection (*n* = 2–3 independent experiments with 4–6 mice per group or per time point per experiment). Data was analysed by two-way ANOVA **p < 0.01 as indicated.From lung tissue, ISG mRNA expression was determined by quantitative PCR at 24 h (*n* = 2 independent experiments with 4–6 mice per group per experiment). NF-κB *p65*^+/−^ mice had significantly reduced levels of COX2 mRNA whilst expressing similar levels of Viperin, PKR and OAS1A mRNA compared to *p65*^+/+^ mice at 24 h. Data was analysed by one way ANOVA *p < 0.05 as indicated.*IFNAR1*^−/−^ had reduced IFN-α protein in BAL at 24–72 h and reduced IFN-λ at 48 h post infection compared to wild-type control mice (*n* = 2–3 independent experiments with 4–6 mice per group or per time point per experiment). Data was analysed by two-way ANOVA ***p < 0.001, **p < 0.01 as indicated.*IFNAR1*^−/−^ mice had similar COX2 mRNA expression compared to wild-type mice however had reduced levels of Viperin, PKR and OAS1A mRNA at 24 h post-infection (*n* = 2 independent experiments with 4–6 mice per group per experiment). Data was analysed by one way ANOVA *p < 0.05 as indicated.NF-κB *p65*^+/+^ and *p65*^+/−^ mice had similar RV1B virus load whilst *IFNAR1*^−/−^ mice had increased virus load compared to wild-type mice at 24 h post infection (*n* = 1 experiment with 4–6 mice per group or per time point). Data was analysed by two-way ANOVA, **p < 0.01 as indicated. ns = not significant, all data are presented as mean ± SEM.

To define the role of p65 *in vivo*, we have used RV infection models using *p65*^+/+^ and *p65*^+/−^ mice on a Bl/6 129 *TNFR1*^−/−^ background. In this model, cellular responses induced by RV were replication-dependent as UV-inactivated virus did not induce airway inflammation (Supporting Information Fig S3). To demonstrate functional *p65* deficiency, we showed that *p65*^+/−^mice expressed less p65 protein and exhibited less p65 activation during RV infection when compared to *p65*^+/+^ controls (Supporting Information Fig S4). To determine if the *TNFR1*^−/−^ background influenced immune responses to RV infection, we performed additional experiments in wild-type Bl/6 129 mice. We found that wild-type Bl/6 129 mice and *TNFR1*^−/−^ *p65*^+/+^ Bl/6 129 mice both induced robust p65 activation, neutrophillic and lymphocytic inflammation, cytokine chemokine and IFN production (Supporting Information Fig S5). Consistent with the HBEC *in vitro* data, p65-deficient mice did not show reduced levels of type I or type III IFN following RV infection *in vivo*. In fact, *p65*^+/−^ mice tended to produce more IFN protein in BAL compared with *p65*^+/+^ mice, and this was significant for IFN-α (Fig 4A). As *p65*^+/−^ mice had intact or even enhanced IFN production, we hypothesized that lung antiviral interferon-stimulated gene (ISG) expression levels would also be comparable between *p65*^+/+^ *and p65*^+/−^ mice whereas ISG expression would be impaired in *IFNAR1*^−/−^ mice, which lack type I IFN receptor signalling. The NF-κB p65-regulated pro-inflammatory enzyme cyclooxygenase (COX-2) was used as a control in these experiments. As expected, RV-infected *p65* deficient mice expressed significantly reduced levels of COX-2 mRNA compared with *p65*^+/+^ mice. In contrast, we observed normal RV-induced Viperin, double-stranded RNA-dependent protein kinase R (PKR) and 2′,5′-oligoadenylate synthetase 1 (OAS1A) expression in *p65*-deficient mice (Fig 4B). To confirm the importance of type I IFN signalling for antiviral responses to RV infection in the mouse model, we performed parallel infection experiments with wild-type (wt) and *IFNAR1*^−/−^ mice and observed that production of type I and type III IFNs was significantly impaired (Fig 4C). In contrast to our observations in *p65*^+/−^ mice, COX-2 gene expression was normal in the lungs of RV-infected *IFNAR1*^−/−^ mice whilst ISG expression was severely impaired (Fig 4D). We next assessed the effect of NF-κB p65 suppression and absence of IFNAR1 signalling on RV load. RV viral (v) RNA increased from 8 to 24 h when it peaked at similar levels in *p65*^+/+^ and *p65*^+/−^ mice, but was significantly increased in *IFNAR1*^−/−^ mice compared wt controls (Fig 4E).

### Lack of type I IFN signalling and reduced p65 expression differentially regulate RV-induced neutrophilic inflammation

**Figure 5:**
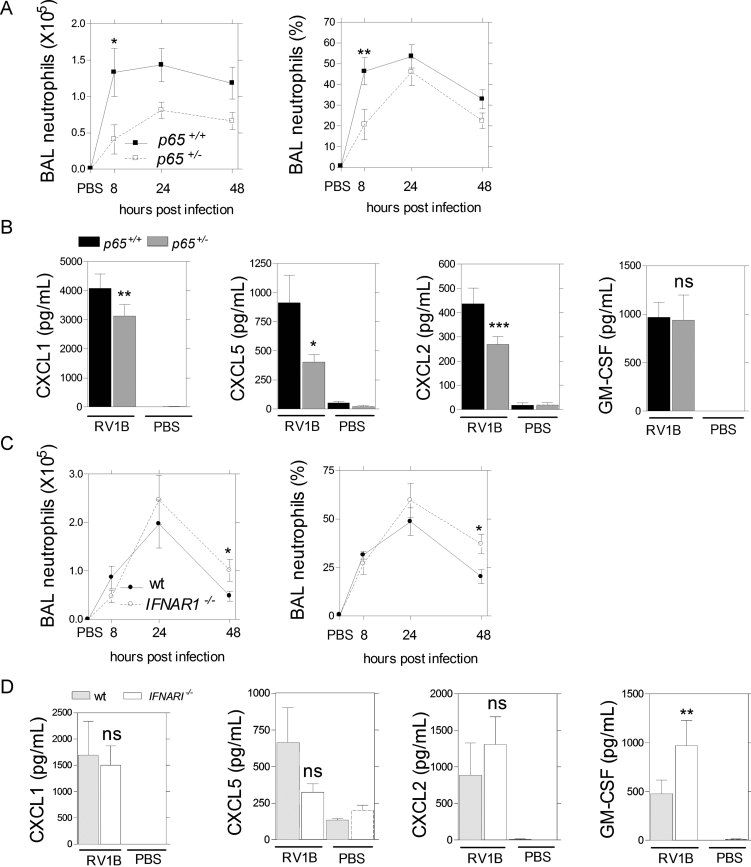
*P65^+/−^* mice have reduced airway neutrophilic inflammation Mice were inoculated with intranasal RV1B or PBS and harvested at various time points. NF-κB *p65*^+/−^ mice had reduced BAL neutrophils (total number and % of BAL cells) at 8 h compared to *p65*^+/+^ mice (*n* = 2–3 independent experiments with 4–6 mice per group or per time point per experiment). Data was analysed by two-way ANOVA **p < 0.01, *p < 0.05 as indicated.At 8 h post infection, NF-κB *p65*^+/−^ mice had reduced BAL CXCL1, CXCL5, CXCL2 but not GM-CSF compared to *p65*^+/+^ controls (*n* = 2 independent experiments with 8–12 mice per group). Data was analysed by one way ANOVA ***p < 0.001, **p < 0.01, *p < 0.05 as indicated.*IFNAR1*^−/−^ mice had increased BAL neutrophils (total number and % of BAL cells) at 48 h (*n* = 2–3 independent experiments with 4–6 mice per group or per time point per experiment). Data was analysed by two-way ANOVA, *p < 0.05 as indicated.At 8 h post infection, *IFNAR1*^−/−^ mice had equal BAL CXCL1, CXCL5, CXCL2 and elevated GM-CSF levels compared to wildtype controls (*n* = 2 independent experiments with 4–6 mice per group per experiment). Data was analysed by one way ANOVA, **p < 0.01 as indicated, ns = not significant, all data are expressed as mean ± SEM.

Lung neutrophilia is associated with disease severity in asthma exacerbations (Message et al, 2008). We therefore investigated regulation of neutrophilic inflammation in *p65*^+/−^ and *IFNAR1*^−/−^ mice. Reduced expression of p65 clearly attenuated RV-induced airway neutrophilia in terms of both total number and percentage of cells in BAL and this was significant at 8 h post-infection (Fig 5A). Impaired neutrophil recruitment in RV-infected p65-deficient mice was associated with reduced induction of the neutrophil-attracting chemokines CXCL1, CXCL2 and CXCL5 at 8 h. In contrast, production of the granulocyte growth and activation factor GM-CSF was not different between *p65*^+/+^ and *p65*^+/−^ mice (Fig 5B). We also investigated the role of IFNAR1 signalling in RV-induced airway neutrophilia. Initially, RV-induced neutrophil recruitment in *IFNAR1*^−/−^ mice was similar to wild-type mice. However, by 48 h significantly increased numbers of neutrophils were observed in the BAL from *IFNAR1*^−/−^ mice (Fig 5C). Consistent with similar kinetics of neutrophil recruitment and in contrast to p65-deficient mice, no differences in neutrophil chemokine production were observed, however, we did observe twofold increased GM-CSF levels in the BAL of *IFNAR1*^−/−^ mice at 8 h (Fig 5D).

### Type I IFN signalling, but not NF-κB p65, is critical for lung lymphocyte recruitment

**Figure 6:**
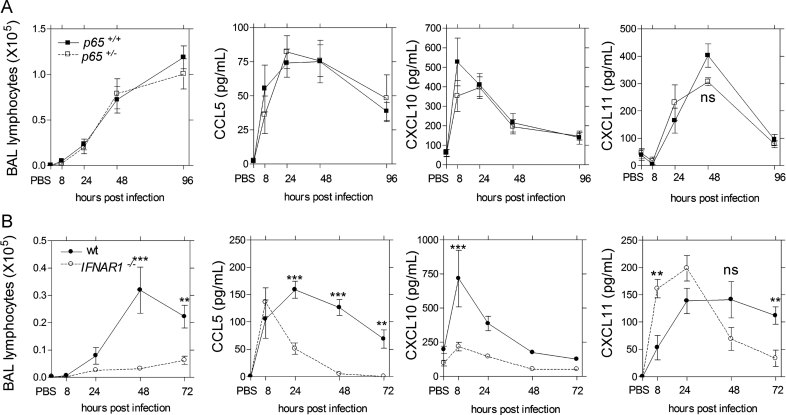
Type I IFN and not p65 are important for lymphocyte recruitment Mice were inoculated with intranasal RV1B or PBS and harvested at various time points. *P65*^+/−^ mice had similar BAL lymphocytes at 8–96 h compared to *p65*^+/+^ mice (*n* = 2–3 independent experiments with 4–6 mice per group or per time point per experiment), and had similar levels of BAL CCL5, CXCL10, and CXCL11 (*n* = 2 independent experiments with 4–6 mice per group or per time point per experiment). Data was analysed by two-way ANOVA, ns = not significant.*IFNAR1*^−/−^ mice had reduced BAL lymphocytes from 48–72 h post infection compared to controls (wt, *n* = 2–3 independent experiments with 4–6 mice per group or per time point per experiment), and reduced BAL CCL5 at 24–48 h, reduced CXCL10 at 8 h elevated CXCL11 at 8 h which was reduced at 72 h (*n* = 2 independent experiments with 4–6 mice per group or per time point per experiment). Data was analysed by two-way ANOVA, ***p < 0.001, **p < 0.01 as indicated, all data are expressed as mean ± SEM.

Recruitment of lymphocytes to the airways is a cardinal feature of the host defence immune response to RV infection (Bartlett et al, 2008; Fraenkel et al, 1995; Message et al, 2008). We observed no differences between *p65*^+/+^ and *p65*^+/−^ mice with respect to lymphocyte numbers or lymphocyte-recruiting chemokines in BAL following RV infection (Fig 6A) indicating that inhibition of p65 would be unlikely to impact on this aspect of host defence. The converse was true for type I IFN signalling. *IFNAR1*^−/−^ mice were severely deficient for lymphocyte recruitment and this was associated with significantly impaired lymphocyte-recruiting chemokine (CCL5, CXCL10 and CXCL11) production in BAL (Fig 6B).

### *IFNAR1*^−/−^ mice exhibit impaired NK- and T-cell responses to RV infection

**Figure 7:**
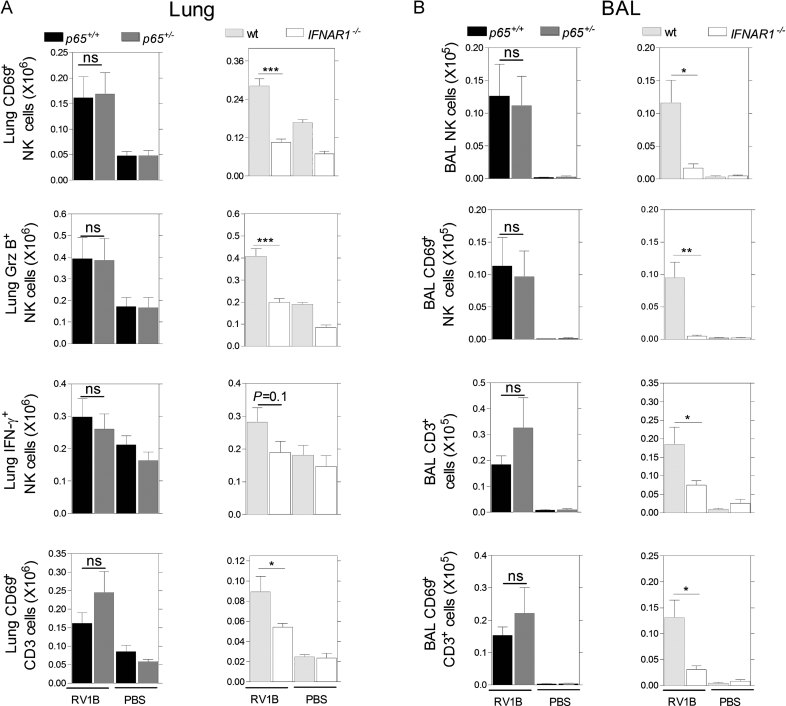
Type I IFN but not p65 are important for antiviral NK- and T-cell responses Mice were inoculated with intranasal RV1B or PBS and harvested at 72 h. Flow cytometry was used to show that *P65*^+/+^ and *p65*^+/−^ mice had similar numbers of lung CD69^+^ lung NK cells, NK cells expressing Grz B and IFN-γ and CD69^+^ CD3^+^ T cells (*left panel*) while *IFNAR1*^−/−^ mice had reduced lung CD69^+^ NK cells and Grz B^+^ NK cells a trend for reduced numbers of NK cells expressing IFN-γ and less CD3^+^ CD69^+^ T cells compared to wildtype (*right panel*; *n* = 2 independent experiments with six mice per group per experiment for both). Data was analysed by one way ANOVA, ***p < 0.001, *p < 0.05 as indicated. ns = not significant.NF-κB *p65*^+/+^ and *p65*^+/−^ had similar numbers of total BAL NK cells, CD69^+^ NK cells, total CD3^+^ T cells and CD69^+^ CD3^+^ T cells, (*left panel*). *IFNAR1*^−/−^ mice had reduced numbers of BAL NK cells, reduced CD69^+^ NK cells, reduced CD3^+^ T cells and CD69^+^ CD3^+^ T cells (*right panel*; *n* = 2 independent experiments with six mice per group per experiment for both). Data was analysed by one way ANOVA, *p < 0.05 as indicated. ns = not significant. All data are expressed as mean ± SEM.

We next assessed the specific cell types that composed the early lymphocyte response to RV by flow cytometry. The details of the flow cytometry analysis are shown in Supporting Information Fig S6. In lung tissue, *p65*^+/−^ mice had similar numbers of RV-induced NK cells that were activated [CD69^+^, granzyme B^+^ (Grz B^+^) or IFN-γ^+^] compared to *p65*^+/+^ controls. Numbers of activated (CD69^+^) T cells were also similar between *p65*^+/−^ and *p65*^+/+^ mice. In contrast, these NK- and T-cell responses were significantly impaired in the lungs of *IFNAR1*^−/−^ mice (Fig 7A). We also analysed NK- and T-cell responses in the BAL and found that *p65*^+/−^ mice had similar numbers of NK, CD69^+^ NK cells, T cells and CD69^+^ T cells to *p65*^+/+^ mice. The *IFNAR1*^−/−^ mice had almost no BAL NK or T cells at 72 h post RV infection. The few NK and T cells that could be detected in the BAL did not exhibit significant up-regulation of CD69 expression (Fig 7B).

## DISCUSSION

We have observed for the first time RV-induced activation of NF-κB p65 in the human bronchial epithelium *in vivo* and have used mouse models of RV infection to demonstrate that NF-κB p65 is a central regulator of RV-induced inflammation in the airways *in vivo*. Furthermore, we provide evidence that suppressing p65 expression, whilst reducing airways inflammation, did not suppress IFN production or antiviral immune responses. NF-κB p65 is therefore identified as an attractive target for development of anti-inflammatory therapies that would not further impair already deficient IFN responses in exacerbations of asthma and chronic obstructive pulmonary disorder (COPD). In doing so, we have also provided clear evidence that responses mediated by type I IFN are critical for antiviral responses to RV *in vivo*. Thus, development of new therapies to replace deficient IFN responses in these conditions is also likely to be of clinical benefit.

Historically, the IFN-β promoter has been thought to be activated via an enhanceosome, requiring the coordinated assembly of IRFs, NF-κB p65 and p50 subunits and the DNA-bending protein high mobility group protein-1 (HMG-1; Du & Maniatis, 1994; Panne et al, 2007). This paradigm has persevered for several years until experiments in the chemically mutated fibrosarcoma P2.1 cell line demonstrated that NF-κB was not required for dsRNA-induced IFN-β transcription (Peters et al, 2002). In these studies, the different subunits of NF-κB were not considered and this observation remained merely an interesting observation in an unusual cell type. More recently, studies in MEFs considered the role of NF-κB subunits including p65 and found that it wasn't required for IFN-β gene transcription in response to various viruses (Wang et al, 2007); yet, a follow-up paper then proposed that NF-κB p65 was crucial for early IFN-β induction before IRF-3 activation was maximal (Wang et al, 2010). These studies highlight that our understanding of IFN-β gene expression during virus infection is still very much incomplete. Importantly, no studies to date have investigated the role of transcription factors in regulating IFN induction by a clinically important virus in its natural host cell, and none have done so *in vivo*.

In light of the current prevailing dogma, our findings that NF-κB p65 is not required for IFN-β induction *in vitro* or the antiviral response to RV *in vivo* at any time point are very surprising. Regarding the more recently discovered antiviral type III IFN-λs, three *in vitro* studies show that their induction is NF-κB-dependent (Onoguchi et al, 2007; Osterlund et al, 2007; Siegel et al, 2011). Studies reported here clearly showed no functional role in IFN-λ regulation for NF-κB p65 *in vitro* or *in vivo*.

To define the role of p65, we have used RV infection models *in vitro* and *in vivo* where p65 expression is substantially reduced by up to 90% (Supporting Information Figs S1 and S4). We chose siRNA knockdown of p65 and an IκB DN as methods to inhibit NF-κB p65; another potential approach could have been pharmacological IKK-β inhibitors (Birrell et al, 2005; Frelin et al, 2003; Kishore et al, 2003). We elected to use molecular approaches over pharmacological inhibition of IKK-β as several reports describe IKK-β mediating IFN production (Chu et al, 1999; Schmolke et al, 2009; Wang et al, 2010), possibly through IRF3 activation (Han et al, 2004). It is possible that NF-κB may simply not be required for IFN-β and IFN-λ gene expression upon RV infection or that p65 has a less important role (Wang et al, 2007, 2010). Further experiments are required to distinguish between p65 acting in either a reduced capacity *versus* being completely irrelevant for IFN transcription. If p65 is required for IFN induction but at a reduced capacity, our data support the notion that p65 inhibition *in vivo* with available inhibitors that achieve substantial but incomplete p65 inhibition (Birrell et al, 2005) could be useful, as they would likely suppress inflammation but not affect IFN expression.

To confirm our *in vitro* studies, we bred *p65*^+/−^ mice for use in an *in vivo* model of RV infection. We first attempted to breed *p65*^−/−^ mice but found these died from bacterial infection before 6 weeks of age despite antibiotic treatment and could not be studied. As p65 deletion is embryonic lethal (Beg et al, 1995), these mice are bred on a *TNFR1*-deficient background. The original report showed that p65-deficient mice had normal p50 expression, although the other NF-κB subunits were not examined. Despite the *TNFR1*^−/−^ background, we still observed robust neutrophil accumulation in the BAL in *p65*^+/+^ *TNFR1*^−/−^ animals, and wild-type Bl/6 129 mice were comparable to *TNFR1*^−/−^ *p65*^+/+^ mice in terms of airway neutrophilia, pro-inflammatory cytokine induction and p65 activation. Despite the obvious effect of partial p65 depletion on neutrophilia, the lack of effect of partial p65 depletion on IFN and ISG induction, NK cells, T cells and RV replication *in vivo* clearly demonstrated that NF-κB p65 was not important for IFN-mediated antiviral immunity to RV. Together, these data strongly suggest that targeted inhibition of p65 *in vivo* will still significantly impact on excessive airway inflammation without interfering with antiviral immunity. Hence, we provide important new evidence that IFN-β and IFN-λ are regulated independently of NF-κB p65 in an *in vivo* model relevant to human disease.

NF-κB p65 activation is associated with inflammation in the airways of asthmatics (Hart et al, 1998). Little is known about RV infection and NF-κB *in vivo*, however, numerous *in vitro* studies have shown that RV infection is also a potent activator of p65, which in turn regulates production of pro-inflammatory cytokines (Funkhouser et al, 2004; Kato et al, 2007; Zhu et al, 1996, 1997). Here, we provide direct evidence in man and mouse that RV infection increases NF-κB activation in the lung. Based on separate studies of RV infection (Bossios et al, 2008) and asthma (Broide et al, 2005; Poynter et al, 2002; Stacey et al, 1997), it has been speculated that RV-induced asthma exacerbations are a consequence of allergen-induced responses and infection interacting to cause increased NF-κB activation and NF-κB-regulated inflammation in the airways. We can now report this to be the case *in vivo* demonstrating enhanced p65 DNA binding activity.

Type I IFN is critical for antiviral immunity, which in turn is essential for efficient control of viral infection and resolution of associated immunopathology. Therapeutic anti-inflammatory approaches that impair innate IFN production may be harmful in asthma, where IFN-mediated antiviral responses are already deficient (Contoli et al, 2006; Wark et al, 2005). We show here that reduced NF-κB p65 expression did not affect antiviral immune responses and had no impact on virus load. RV infection of mice, which lacked the type I IFN receptor, had completely contrasting effects. Severely impaired antiviral responses were associated with increased viral load and prolonged neutrophilic inflammation. Showing that RV and allergen interact to enhance NF-κB p65 and airways inflammation, and by comparing RV infection in p65 gene-deficient mice has, for the first time, provided clear evidence that suppressing p65 is an attractive anti-inflammatory approach for asthma exacerbations that will not impair host antiviral defence. Furthermore, we definitively show the role of type I interferon in RV infection *in vivo* and its importance in controlling RV replication and RV driven inflammation, important endpoints in human exacerbations of asthma.

Previous studies have shown that RV infection evokes IFN production from human lung bronchial epithelial cells (Khaitov et al, 2009; Slater et al, 2010) and IFN-β treatment stimulates antiviral resistance in RV-infected bronchial epithelial cells (Cakebread et al, 2011; Wark et al, 2005). Only one study has linked deficient production of RV-induced antiviral IFN with RV-induced disease severity *in vivo* (Contoli et al, 2006). From such studies, the importance of IFN *in vivo* has been inferred, however, causal relationships cannot be definitively established by correlations alone. Here, we have demonstrated that IFNAR1 signalling is required for amplification of IFN and maximal type III IFN protein expression. These data clearly demonstrate the importance of IFN-induced IFN for amplification of virus-induced IFN in antiviral immunity. For type III IFN, in the absence of type I IFN signalling, expression was deficient later (48 h) in the infection presumably because viral RNA levels had dropped by this time and there was no type I IFN signalling to amplify IFN-λ production. The temporal responses of type I IFN and IFN-λ in *IFNAR1*^−/−^ mice highlight the critical role IFN production plays in a positive feedback loop, which is important in reducing peak virus load and virus-induced airways inflammation.

Using recently developed mouse RV infection models, we and others have demonstrated RV replication in mouse lung tissue using detection of total or negative-strand viral RNA (Bartlett et al, 2008; Newcomb et al, 2008) or RV proteins in bronchial epithelium (Newcomb et al, 2008). However, the current mouse models do exhibit limited permissiveness for RV replication such that even in *IFNAR1*^−/−^ mice (where antiviral responses are attenuated and virus titres are significantly higher than that in controls) virus clearance still occurs. Nonetheless, this model clearly identifies type I IFN signalling (and not NF-κB p65) as being important for controlling RV replication and inducing anti-viral immunity.

This study is the first to distinguish molecules required for either RV-induced inflammation or anti-viral immune responses *in vivo*. A previous study investigated the importance of MDA5 and TLR3 for lung interferon and pro-inflammatory cytokine gene expression and RV clearance (Wang et al, 2011). Analyses were almost entirely limited to lung mRNA expression and anti-viral cellular responses were not reported. RV-infected MDA5-knockout mice did exhibit reduced IFN production, however, there was not a clear difference in viral replication. Wang et al also studied TLR3-knockout mice reporting absence of TLR3 has no effect on RV-induced type I or type III IFN production and virus clearance. This result is puzzling since studies by this group (Wang et al, 2009) and ourselves (Slater et al, 2010) have reported that TLR3 signalling is required for RV-induced IFN expression *in vitro* by primary HBECs. Our *in vitro* and *in vivo* models do support the conclusion that NF-κB p65 is not important for RV-induced IFN and anti-viral responses, yet is critical for RV-induced inflammation.

The absence of IFNAR1 almost completely abolished RV-induced lymphocyte recruitment and lymphocyte chemokines. RV infection has been shown to directly induce expression of lymphocyte chemokines *in vitro* (Zaheer & Proud, 2010). For CXCL10 this has been shown to be directly regulated by IFN-β (Chen et al, 2006). Given the profound effect of IFNAR1 signalling on chemokine production and subsequent lung lymphocyte recruitment, we examined CD3^+^ lymphocytes and NK cells since they were likely to constitute the dominant early antiviral lymphocyte response in the lungs (Gazit et al, 2006; Taylor et al, 1985). *IFNAR1*^−/−^ had reduced lung tissue and BAL NK-cell responses. Very little is known about the role NK cells play following RV infection other than the observation that human PBMC-derived NK cells are activated upon infection (Gern et al, 1996; Hsia et al, 1990; Levandowski & Horohov, 1991). No studies to date have examined RV-induced NK-cell responses in the lung. We demonstrate that RV infection elicits a type I IFN-dependent NK-cell response (involving up-regulation of CD69, Grz B and IFN-γ) in the lung.

In conclusion, we report that inhibition of NF-κB p65 suppresses airway inflammation but does not impact on host antiviral defence suggesting that while crucial for inflammation, p65 is not critical for IFN gene expression and is therefore an attractive therapeutic target for RV-induced asthma exacerbations. In contrast, we show that type I IFN signalling is critical for effective anti-viral immunity and therefore replacement of deficient IFN in asthma exacerbations is a further attractive therapeutic approach.

## MATERIALS AND METHODS

### Cells and viruses

HBECs from non-asthmatic, non-smoking individuals were obtained from a commercial source (Clonetics, Wokingham, UK), and cultured in BEGM with supplements according to the suppliers recommended protocol (Clonetics). Unless otherwise stated, all data was derived from experiments from three different HBEC sources. BEAS-2B cells (European Collection of Cell Cultures) were cultured in RPMI with 10% FCS (Invitrogen, Paisley, UK). Major group RV16 and minor group RV1B were grown in HeLa cells. For *in vivo* use, RV1B was purified as previously described (Bartlett et al, 2008).

### Plasmids and siRNA

ΔRIG-I, PEF-BOS and IFN-β Luc-125 bp were gifts from T. Fujita (Kyoto, Japan). IL-6-Luc, containing a 651 bp fragment of the human IL-6 promoter was obtained from R. Panettieri. ΔTRIF and pUNO1 were purchased from Invivogen (Nottingham, UK). The IκBα DN mutant was purchased from Clontech, and pcDNA3.1 from Stratagene. IFN-λ1.3 Luc was produced by cutting a 371 bp fragment from the parental 1kB IFN-λ1 promoter (Osterlund et al, 2007) and subcloned into pGL3 (Promega, Madison, USA). All siRNA including control siRNA (specific for luciferase) were purchased from Dharmacon (Lafayette, CO, USA). SiRNA to each target were a pool of four individual siRNA, while the control siRNA was a single individual siRNA specific for an irrelevant gene (firefly luciferase).

### Transient transfections of HBECs and BEAS-2B cells with siRNA or plasmid DNA

HBEC cells were cultured to 80% confluence in 12 well plates and transfected with 100 nM specific siRNA or control siRNA for 24 h prior to infection with RV1B. All siRNA experiments were performed and validated as previously described (Slater et al, 2010). HBECs were cultured to near confluence in 12 well plates and then transfected with 0.8 µg per well of a minimal NF-κB reporter (Agilent Technologies, Stockport UK), and 0.2 µg of Renilla plasmid (Promega) for 5 h. All transfections utilized Lipofectamine 2000 (Qiagen, Crawley, UK) according to the manufacturers recommended protocol. Complexes were removed, medium replaced and cells left for 24 h. Cells were infected with RV1B and lysates taken 24 h post infection. Alternatively, HBECs were transfected with 0.65 µg per well of ΔRIG-I, pEF-BOS control vector (Yoneyama et al, 2004) or constitutively active TRIF (ΔTRIF, Invivogen) or the pUNO1 control vector (Invivogen), 0.25 µg minimal NF-κB reporter and 0.1 µg Renilla as above. BEAS-2B cells were transfected with 0.1 µg per well of IκBα DN (Clontech), pcDNA3.1 as empty vector control (Stratagene) along with 0.7 µg per well of the IL-6 Luc, IFN-β or IFN-λ1 Luc promoter, with 0.1 µg Renilla (Promega). Transfections used Superfect (Qiagen), according to the manufacturer's recommended protocol. Lysates were harvested 24 h post transfection, and luciferase measured according to the dual luciferase protocol (Promega) on a Berthold luminometer (Berthold, UK). Data were expressed as ratios of firefly over Renilla luciferase.

### Infection of HBECs

HBEC cells were placed in BEGM without serum or additives and then infected with RV1B or treated with medium for 1 h with shaking. Medium was replaced and lysates or supernatants harvested at relevant time points.

### Immunostaining of human bronchial biopsies

Bronchial biopsies were obtained from five non-allergic, non-asthmatic, non-smoking individuals in a previous *in vivo* challenge study with RV16 (Message et al, 2008). All subjects gave informed consent and the study was approved by St Mary's National Health Service Trust Research Ethics Committee. Bronchial biopsies were embedded in Tissue Tek II OCT (Miles Scientific, Naperville, IL), frozen within 15 min in isopentane pre-cooled in liquid N_2_ and stored at −80°C. Frozen samples were then oriented and 6 µm thick cryostat sections were cut for immunohistochemical staining and analysis using light microscopy, as previously described (Di Stefano et al, 2002). We quantified the cells with at least a portion of the nucleus clearly seen close to the area of immunopositivity (Jeffery et al, 2003). The number of positive cells was enumerated, with a cell being scored positive if 50% or more of their nucleus stained positive for NF-κB p65.

### Mice and RV infections

All work involving mice was in accordance with legislation outlined by the Home Office, UK. *TNFR1*^−/−^ NF-κB *p65*^+/−^ mice and *TNFR1*^−/−^ NF-κB *p65*^+/+^ on Bl6/129 background were bred as previous described (Quinton et al, 2007). *IFNAR1*^−/−^ mice on C57/Bl6 background were bred in house, female Balb/c, Bl/6 129 and C57/Bl6 controls were purchased from Harlan (Harlan-Sprague-Dawley, UK). The mouse RV-infection and exacerbation of allergic airways inflammation models been described previously (Bartlett et al, 2008).

### BAL cell preparation and analysis by cytology

Cells were pelleted by brief centrifugation, resuspended in ACK buffer to lyse red cells, washed with PBS and resuspended in 1 ml RPMI with 10% FCS. Cells were stained with Quik Diff (Reagena) for differential counts.

### Electrophoretic mobility gel shift Assay (EMSA) and assessment of nuclear p65 activation

The levels of NF-κB in the nuclear fraction were assessed by EMSA as described previously (Birrell et al, 2005). Nuclear NF-κB p65 activation in a model of RV exacerbation of allergic airways inflammation was also assessed using a commercial kit (TransAM p65 DNA binding assay, Active Motif, La Hulpe, Belgium), using 20 µg per well of nuclear protein and the manufacturer's recommended protocol.

**The paper explained**

PROBLEM:

Asthma is a disease affecting 300 million people worldwide. The majority of the morbidity and mortality of asthma is associated with asthma exacerbations, a form of the disease, which responds poorly to conventional asthma treatments. Respiratory infections by human rhinovirus account for most asthma exacerbations, with virus-induced lung inflammation and lung damage directly related to loss of lung function. Asthmatics are also deficient in production of type I and type III interferons, anti-viral cytokines required for defence against rhinovirus, although the role interferon plays in rhinovirus infection *in vivo* is incompletely elucidated. Importantly, several decades of research have suggested that NF-κB p65 is required for both virus-induced inflammation and interferon production; however, detailed investigations using virus–host combinations relevant to human disease are lacking.

RESULTS:

*In vitro*, in primary bronchial epithelial cells and cell lines, inhibition of NF-κB p65 resulted in inhibition of rhinovirus-induced pro-inflammatory cytokines, however, type I and type III interferons were not inhibited*. In vivo*, *p65*^+/−^ mice had reduced neutrophils and pro-inflammatory cytokines but had intact anti-viral responses including interferons and intracellular anti-viral enzymes. In parallel studies, the importance of interferon in the host response to rhinovirus *in vivo* was revealed using interferon-α receptor 1-deficient mice (*IFNAR1*^−/−^), which had intact inflammatory responses but reduced anti-viral immunity in the form of type III interferon induction, anti-viral intracellular enzymes, lung T- and NK-cell recruitment and greater virus loads when compared to control mice.

IMPACT:

The data highlight the fact that in rhinovirus infection, protective anti-viral responses can be uncoupled from harmful, pro-inflammatory responses. Targeting NF-κB p65 may be a reasonable therapeutic strategy for asthma exacerbations, specifically addressing inflammation without compromising beneficial anti-viral immunity, which is sub-optimal in asthmatics. The data also question several years of research supporting the role of NF-κB p65 in type I interferon and type III interferon gene expression and that interferon is crucial to the control of rhinovirus infection *in vivo*.

### RNA extraction, cDNA synthesis and Quantitative PCR

Total RNA was extracted from HBECs (RNeasy kit, Qiagen), and 2 µg was used for cDNA synthesis (Omniscript RT kit, Qiagen). Total RNA was also extracted from the apical lobe of the mouse lung, and placed in RNA later (Qiagen), prior to RNA extraction and cDNA synthesis (as above). Quantitative PCR was carried out using specific primers and probes for each gene (Supporting Information Table SV). Reactions were analysed using an ABI 7500 TaqMan, (ABI Foster City, CA, USA). Each gene was normalized to 18S rRNA, and expressed as copies per microlitre cDNA reaction using a standard curve based on amplification with plasmid DNA. For siRNA experiments in HBECs, copy number was expressed as a % of copy number *versus* RV1B infected and transfected with control siRNA.

### ELISA analysis of BAL cytokine production

ELISAs for mouse IFN-α, IFN-β, CXCL1, CXCL2, CXCL5, GM-CSF, CXCL11, CXCL10, TNF-α, IL-1β, IL-6, CCL5, CCL11, CCL17, CCL22, CCL24 and IFN-λ (IL-28) were from R&D Systems (Abingdon, UK). All ELISAs were used according to the manufacturer's recommended protocol.

### SDS–PAGE and Western Blotting

Western blotting, was performed as previously described (Slater et al, 2010), using the following primary antibodies; NF-κB p65, (0.2 µg/ml, Santa Cruz Biotechnology, Inc., CA, USA), IRF1 (1.2 µg/ml, Abnova Corporation, Taipei, Taiwan), IRF3 (0.2 µg/ml, Santa Cruz Biotechnology, Inc.), IκBα (0.5 µg/ml, Santa Cruz Biotechnology, Inc.), α-tubulin (1 µg/ml, Santa Cruz Biotechnology, Inc.), or β-actin (1 µg/ml, Biovision, Mountain View CA, USA).

### Flow cytometry

Lungs were crudely dissociated using the GentleMACS™ tissue dissociator (Miltenyi Biotech, Germany) and digested by incubating at 37°C in buffer containing 1 mg/ml collagenase Type XI and 80 units/ml Bovine Pancreatic DNase Type IV (both Sigma–Aldrich Dorset, UK). Single cell suspensions were attained by further GentleMACS™ dissociation, red cells were lysed with ACK buffer and cells were filtered through a 100 µm cell strainer. For IFN-γ and Grz B expression analyses by intracellular cytokine staining (ICS), lung cells were stimulated for 3 h with PMA (50 ng/ml) and ionomycin (500 ng/ml), (Sigma–Aldrich) in the presence of monensin (Golgi block, BD Biosciences. Lung and BAL cells were stained with Live/dead fixable dead cell stain kit (Invitrogen) with anti-mouse CD16/CD32 (FC block, BD Biosciences). Cell surface markers analysed: CD3e (clone 500A2), NK1.1 (clone PK136) and CD69 (clone H1.2F3; all BD Biosciences). For ICS, cells were permeablized with 0.5% saponin and stained for IFN-γ (clone XMG1.2, BD Biosciences) and Grz B (clone GB12, Invitrogen). Data were acquired using a nine colour CyanADP flow cytometer and Summit v4.3 software (both Dako Ely, UK). Analysis was also performed using Summit v4.3 software.

### Statistical analysis

All *in vitro* experiments were performed 4–6 times, all data expressed as mean ± SEM. Experiments using siRNA or transfection with plasmids were analysed by one-way ANOVA and Bonferroni's multiple comparison test. For differences between two groups, a student's *t*-test was employed with *p* < 0.05 taken as significant. Staining of human bronchial epithelium for p65 was analysed by using the *t*-test, *p* < 0.05 taken as significant. Experiments in the mouse model involved 4–6 animals per group, in up to three independent experiments. Data were analysed using two-way ANOVA and Bonferroni's multiple comparison test. All statistics were performed using Graph Pad Prism 4 software, with *p* < 0.05 taken as significant.

## Author contributions

All experiments were conceived by MRE, NWB, LS and SLJ; LS, JJH and MRE performed *in vitro* work, with advice from SVK; NWB performed *in vivo* mouse model studies, JPM provided NF-κB *p65*^+/−^ mice and assisted with experimental design; NG performed flow cytometery analysis on lung and BAL samples; DLC and NWB performed gel shift studies on mouse lung samples with advice from MB. Clinical samples from experimental RV infections were from a study performed by SDM and PM, with assistance from TK and JA; JZ, PC and GC performed immunohistochemistry of bronchial biopsies for nuclear p65 with advice from AP; MRE, NWB and SLJ wrote the manuscript.

## Supplementary material

Detailed facts of importance to specialist readers are published as ”Supporting Information”. Such documents are peer-reviewed, but not copy-edited or typeset. They are made available as submitted by the authors.
