# Supplementary material for: Defining critical roles for NF-κB p65 and type I interferon in innate immunity to rhinovirus
Source: EMBO Mol Med. 2012 Nov 14;4(12):1244–60. doi: 10.1002/emmm.201201650 (PMC3531601; doi:10.1002/emmm.201201650)
Supplement: Supplementary file 1 [file emmm0004-1244-SD1.pdf]

EMBO Molecular Medicine

Manuscript EMM-2012-01650

Defining critical roles for NF- B p65 and type I interferon in innate immunity to rhinovirus

Nathan W. Bartlett, Louise Slater, Nicholas Glanville, Jennifer J. Haas, Gaetano Caramori, Paolo Casolari, Deborah L. Clarke, Simon D. Message, Julia Aniscenko, Tatiana Kebadze, Jie Zhu Patrick Mallia, Joseph P. Mizgerd, Maria Belvisi, Alberto Papi, Sergei V. Kotenko, Sebastian L. Johnston and Michael R. Edwards

Corresponding author: Michael Edwards, Imperial College London

Review timeline:

Transfer date:	13 June 2012
Editorial Decision:	10 July 2012
Revision received:	07 September 2012
Editorial Decision:	17 September 2012
Revision received:	19 September 2012
Accepted:	20 September 2012

Transaction Report:

(Note: With the exception of the correction of typographical or spelling errors that could be a source of ambiguity, letters and reports are not edited. The original formatting of letters and referee reports may not be reflected in this compilation.)

1st Editorial Decision 10 July 2012

Thank you for the submission of your manuscript "Defining critical roles for NF- B p65 and type I interferon in innate immunity to rhinovirus". We have now heard back from the three referees whom we asked to evaluate your manuscript. You will see that they find the topic of your manuscript potentially interesting but they feel that the data need to be strengthened which should be addressed in a major revision of the manuscript.

In particular, Reviewer #1 highlights that, in addition to a number of control experiments, the use of an NFkB inhibitor to corroborate certain key findings would strengthen the manuscript. In addition, Reviewer #3 points out that the specific involvement of p65 in the response to virus infection should be substantiated.

Given the balance of these evaluations, we feel that we can consider a revision of your manuscript if you can convincingly address the issues that have been raised within the time constraints outlined below.

Revised manuscripts should be submitted within three months of a request for revision. They will otherwise be treated as new submissions, unless arranged otherwise with the editor.

I look forward to seeing a revised form of your manuscript as soon as possible.

Yours sincerely,

Editor
EMBO Molecular Medicine

***** Reviewer's comments *****

Referee #1 (Comments on Novelty/Model System):

The topic is of great interest clinically. The authors propose a dichotomy between pro-inflammatory and antiviral responses during RV-induced asthma exacerbations that can guide future drug development. Unique animal model of RV infection and biopsies from RV-challenged human volunteers (ethics for the later not well described). Experiments overall well controlled. Addition of an NF-kB inhibitor to confirm p65+/- mouse data would further strengthen the manuscript. Perhaps clarity can be improved.

Referee #1 (Other Remarks):

Viral infections of the respiratory track, especially those involving rhinoviruses (RV), are the most common cause of severe exacerbations of asthma and their treatment constitutes a great unmet medical need. The study by Bartlett et al. now reports that it is possible to suppress RV-induced airway inflammation while sparing antiviral responses and reduction of viral load in vivo. Using human bronchial epithelial cells and p65+/- mice, the authors show that p65 NF-kB is essential for the exacerbation of allergic airway inflammation (neutrophilia) upon RV infection but is not required for the induction of type I/III IFNs, the infiltration of NK cells or lymphocytes, or the clearance of RV from the lung. Rather, they show that these later events are dependent on type I IFN signaling, and suggest a dichotomy in the molecular pathways that control pro-inflammatory vs antiviral responses following RV infection. Overall, this is an important and well controlled study. It exhibits significant translational potential in terms of future drug development for viral exacerbations of asthma.

However, the following issues need to be addressed as detailed below:

1. The fact that p65+/- mice need to be kept in a TNRF1-/- is a limitation, although a difficult one to get around. Confirmation of the main findings of this work with a small molecule NF-kB inhibitor (e.g. bortezomib or other proteasome inhibitors) would certainly strengthen the study.

2. In Fig.1, the kinetics of NF-kB activation upon RV infection in both HBECs and mouse lung are somehow slow. How do the authors explain that? Could NF-kB activation be secondary to RV-induced cytokines that subsequently trigger NF-kB such as IL-1 or TNF? As shown in Fig.2, IL-1 is certainly triggered by RV.

3. Is NF-kB p65 similarly activated by RV in 129/Bl6 mice, the actual background of p65+/+ and p65+/- mice?

4. In p.8-9, the authors use the expression "when compared to allergen challenge alone (UV-RV1B-OVA)". However, this group does not involve "allergen challenge alone", it also has UV-inactivated RV. This needs to be corrected.

5. In p65+/- mice, WB levels of other NF-kB subunits (e.g. p50, relB, c-rel) need to be checked. If their expression is also affected (positively or negatively), that needs to be mentioned and discussed.

6. Have the authors confirmed that p65 siRNA is not also depleting other NF-kB subunits or have they confirmed the data with individual p65 siRNAs instead of the pool of four? The same applies for IRF3.

7. Which of the CC and CXC chemokines are NF-kB-dependent? Is there evidence that any of these have NF-kB sites in their promoters or require NF-kB for their expression?

8. Important information and control experiments presented for the first time as Supplemental Figures in the Discussion appear somehow misplaced. These should be presented earlier on together with the main Results. For example, it's important for the reader to know to what extent NF-kB p65

is deficient in p65+/- mice.

9. Discussion could be shortened and made more easy to read.

10. In some cases (e.g. Fig. 4A, 4E, 5A, 6A etc), it is very difficult to distinguish between the labels for p65+/- and p65+/+ mice labels. Please correct.

11. A reference to the Ethics of the experiment in human patients/bronchial biopsies should be made.

Referee #2:

Subtypes of NF- B are essential transcription factors to regulate inflammatory responses to pathogens. Here Bartlett et al demonstrate the functional role of NF- B p65 in rhinovirus (RV) infection. Inhibition of NF- B p65 using siRNA-mediated knockdown did not alter the RV-induced IFNs but reduced the production of inflammatory cytokine and chemokines such as CCL5, CXCL8 and CXCL5. Consistent with this, I B DN expression decreased IL-6 induction but not IFN- and 1. Utilizing p65 heterozygous mice, whose expression level of p65 is much reduced compared to wild type, they clearly showed the same tendency in in-vivo RV infection. Neutrophilic inflammation and production of chemokines were impaired in p65 heterozygous mice, while IFN induction and virus loads were unaffected. In summary, they provide a new insight into RV infection and suggest a therapeutic potential of NF- B p65 for the treatment of RV-induced airway diseases.

Minor comment:

They mention about p65 deficient mice in page 10: "Consistent with HBEC in vitro data p65 deficient mice did not show reduced levels of type I or type III IFN following RV infection in vivo...."

It would be nice to include these data or provide a proper reference for this.

Referee #3:

In this manuscript Bartlett et al examined the role of NF-kB p65 in type I interferon production after Rhinovirus infection. The authors discovered that RV infection induces p65 activation but p65 is not required for IFN-b or IFN-lambda expression. However, they found that p65 is required for airway inflammation. This is a potentially interesting observation since inhibitors of p65 would not affect the antiviral response but could diminish airway inflammation.
The role of NF-kB in IFN-b activation is debated for the last years and many conflicting reports appear in the literature. The current picture says that p65 is required for the early antiviral response and before the entry of IRF3 in the picture. This issue has not been addressed in the current manuscript experimentally by carrying out detailed time-courses. Furthermore some of the biochemical data are not of sufficient quality to fully justify the authors' conclusions. For example, the quality of Figure 1A should be improved and labelled appropriately to indicate nuclear activation and the EMSA should be shown in Fig. 2A together with the appropriate NF-kB subunit identification experiments (antibody super shift experiments) to substantiate the involvement of p65 in the NF-kB complexes formed after virus infection. In other words, it could be that p65 together with other Rel proteins are co-induced and could replace each other. Finally, the paper jumps from human to mouse cells and then back to human and then back to mouse models to substantiate the authors' conclusions. Although these are important steps to validate the clinical role of components it appears that generate a lot of confusion for molecular-mechanistic studies since it could imply species-specific variability. In summary, a more streamlined version of the manuscript could be more informative and interesting for the general audience.

1st Revision - Authors' Response 07 September 2012

Reviewer #1

1. The fact that p65+/- mice need to be kept in a TNRF1-/- is a limitation, although a difficult one to get around. Confirmation of the main findings of this work with a small molecule NF-kB inhibitor (e.g. bortezomib or other proteasome inhibitors) would certainly strengthen the study.

Response: We thought that using a proteasome inhibitor *in vivo* would be difficult to optimise in the time frame given and produce results that may be difficult to interpret. In support, previous studies have shown conflicting results using bortezomib *in vivo*, bortezomib causes apoptosis (Nakata Int J Oncol, 2011) and ER stress which may have various affects on the immune system and inflammation unrelated to NF-kB function, including effects on T cell and B cell responses (Lang J Immunol, 2010;), IFN-gamma production (Yanaba J Leuk Biol, 2010). Bortezomib has also caused increased inflammation in mice infected with RSV (Lupfer Toxicology, 2010). In *ex vivo* culture models, increased induction of inflammatory mediators have also been observed (Cullen Biochem Pharmacol, 2010; Karabela, Carcinogenesis, 2012). Based on this evidence, we concluded that experiments with bortezomib may not help us in clarifying the contributions of TNFR1 deficiency in the RV infection model.

We have therefore assessed RV induction of NF-kB p65 activation and NF-kB inducible cytokines and chemokines in wildtype Bl/6 129 mice. We now show that *TNFR1*$^{-/-}$ is not a limitation to the study of RV infection *in vivo*, as we can report that NF-kB p65 activation and RV induction of IFNs, pro-inflammatory cytokines and chemokines in Bl/6 129 wildtype mice is very similar to Bl/6 129 *TNFR1*$^{-/-}$ *p65*$^{+/+}$ mice, with both groups of mice having robust responses to RV. Activation of p65 and neutrophil chemokines were also very similar. We feel that any differences are small and likely due to different batches of virus used, and don't really support the idea that *TNFR1*$^{-/-}$ mice are fundamentally different to wildtypes in response to RV. We therefore conclude that our results are not confounded by the *TNFR1*$^{-/-}$ background. This new data has been added to the supplementary data online as Fig S5.

2. In Fig.1, the kinetics of NF-kB activation upon RV infection in both HBECs and mouse lung are somehow slow. How do the authors explain that? Could NF-kB activation be secondary to RV-induced cytokines that subsequently trigger NF-kB such as IL-1 or TNF? As shown in Fig.2, IL-1 is certainly triggered by RV.

Response: In HBECs, we see IkB degradation noticeably by 8h, but it is likely that IkB degradation occurs even earlier, however additional time may be taken to see a decrease in the net effect on total cellular IkB. Our western blot measures total IkB, not just IkB attached to p65, which may be reduced earlier but is not detected initially by western blot. We have repeated this experiment in BEAS-2B cells and saw noticeable IkB degradation at 6h and more so at 8h, generally consistent with our data in Fig 1. Small differences could be due to differences between HBECs and BEAS-2B cells or batch variability in the virus stock used. Overall the 8h decrease in IkB degradation *in vitro* is consistent with the induction of pro-inflammatory genes *in vitro*, which is seen at approx. 12hours and peaks at 24h. In Bl/6 129 mice, we performed p65-DNA binding studies over time. p65 DNA binding is evident at 4h, peaking at 8h but still up at 24h compared to uninfected mice. This is consistent with the EMSA data in Fig1, which shows NF-kB-DNA binding evident at 8h and peaking at 24h. Fig 1 now contains the original EMSA data and the confirmation of p65 activation by p65-DNA binding studies.

Overall we feel that NF-kB DNA binding observable by 4-8h is also consistent with the production of cytokines and attraction of neutrophils, peaking at around 24h. While cytokines like TNF-a and IL-1b most likely give additional NF-kB activation, the early activation is likely the direct recognition of virus RNA by pattern recognition receptors etc.

3. Is NF-kB p65 similarly activated by RV in 129/Bl6 mice, the actual background of p65+/+ and p65+/- mice?

Response: As mentioned above we have performed a time course study in BL/6 129 mice (background of *TNFR1*$^{-/-}$ & *p65*$^{-/-}$). We have assessed NF-kB p65 DNA binding and found the levels

to be comparable to that of the *TNFR1*$^{-/-}$ mice. This data is now in presented in supplementary data Figure S5.

4. In p.8-9, the authors use the expression "when compared to allergen challenge alone (UV-RV1B-OVA)". However, this group does not involve "allergen challenge alone", it also has UV-inactivated RV. This needs to be corrected.

Response: We have amended this section as suggested by the reviewer to:

"....observing that live RV infection during allergen challenge (RV1B-OVA) increased NF-kB p65 DNA binding in lung nuclear protein extracts when compared to live infection alone (RV1B-PBS) and allergen challenge together with inactivated RV (UV-RV1B-OVA) (Fig 2A). RV-infection alone and OVA-challenge with UV-RV1B...."

5. In p65+/- mice, WB levels of other NF-kB subunits (e.g. p50, relB, c-rel) need to be checked. If their expression is also affected (positively or negatively), that needs to be mentioned and discussed.

Response: The *p65*$^{-/-}$ and *p65*$^{+/-}$ mice have been well studied previously, and the affects of p65 deficiency on other Rel family members has not been readily reported. The original report (Beg, Nature 1995) saw no affect on p50 activation in cells from *p65*$^{-/-}$ mice. We find it difficult to believe that *p65*$^{+/-}$ mice would have deficiencies on other Rel family members based on this data. Furthermore, p65 (chromosome 19) is encoded by a different chromosome compared with other Rel family members with the exception of p52. The location of p65 is also quite distal from p52 on chromosome 19. It is therefore quite difficult to conceive that interruption of the p65 gene by insertional inactivation would affect genes at distant sites.

We have entered the statement in the discussion: *"The original report showed that p65 deficient mice had normal p50 expression (Beg et al 1995), although the other NF-kB subunits were not examined."*

6. Have the authors confirmed that p65 siRNA is not also depleting other NF-kB subunits or have they confirmed the data with individual p65 siRNAs instead of the pool of four? The same applies for IRF3.

Response: We have not individually tested each siRNA in the pool of four for the p65 or IRF3 siRNA, arguing that the pool is more likely to give better knockdown and have less chance of producing off target affects. We have however performed additional assays to confirm the effects of p65 and IRF3 siRNA on related transcription factors. We found that p65 siRNA did not significantly affect p50, p52, RelC or RelB mRNA levels compared to untransfected or cells transfected with a control siRNA. Likewise we found that IRF3 siRNA had no significant affect on IRF1, IRF5 IRF7 or IRF9 mRNA. One exception was that IRF3 siRNA increased IRF1 and IRF9 mRNA in one experiment, increasing the overall mean. This was not seen in subsequent repeats, so overall these differences are not significant. This data has been added as supplementary data Table SIV.

7. Which of the CC and CXC chemokines are NF-kB-dependent? Is there evidence that any of these have NF-kB sites in their promoters or require NF-kB for their expression?

Response: Yes most of these cytokines/chemokines are positively regulated by NF-kB family members and have Rel binding sites in their promoters. By searching the literature and searching the Transcriptional Regulatory Element Database (TRED) rulai.cshl.edu/TRED/, it is clear that the human chemokines CXCL1, CXCL8, CXCL5, and CCL5, CXCL10 are all Rel dependent. For the mouse, TNF-a and the human CXC homologs CXCL1, CXCL2, CXCL5 and CXCL10 are also Rel dependent. COX-2 is also Rel A dependent (Mempel JID, 2003).

8. Important information and control experiments presented for the first time as Supplemental Figures in the Discussion appear somehow misplaced. These should be presented earlier on together with the main Results. For example, it's important for the reader to know to what extent NF-kB p65 is deficient in p65+/- mice.

Response: We have amended this as suggested by the reviewer. In the results section were the animal model is introduced and the data with p65 deficient mice first described, we have entered the statements:

"To define the role of p65 in vivo, we have used RV infection models using $p65^{+/+}$ *and* $p65^{+/-}$ *mice, on a Bl/6 129* $TNFR1^{-/-}$ *background. In this model, cellular responses induced by RV were replication dependent, as UV-inactivated virus induced no airway inflammation (supplementary information Fig S3). To demonstrate functional p65 deficiency we showed that* $p65^{+/-}$*mice expressed less p65 protein and exhibited less p65 activation during RV infection when compared to* $p65^{+/+}$ *controls (supplementary information Fig S4). To determine if the* $TNFR1^{-/-}$ *background influenced immune responses to RV infection we performed additional experiments in wildtype Bl/6 129 mice. We found that wildtype Bl/6 129 mice and* $TNFR1^{-/-}$ $p65^{+/+}$ *Bl/6 129 mice both induced robust p65 activation, neutrophillic and lymphocytic inflammation, cytokine, chemokine and IFN production (supplementary information Fig S5)."*

9. Discussion could be shortened and made more easy to read.

Response: We have shortened the discussion as suggested by the reviewer, reducing sections on pages 15-19. The discussion is now 6 pages down from 7.5.

10. In some cases (e.g. Fig. 4A, 4E, 5A, 6A etc), it is very difficult to distinguish between the labels for p65+/- and p65+/+ mice labels. Please correct.

Response: We have amended this as suggested by the reviewer, and have made these labels larger.

11. A reference to the Ethics of the experiment in human patients/bronchial biopsies should be made.

Response: We have amended this as suggested by the reviewer. We have inserted the statement "*All subjects gave informed consent and the study was approved by St Mary's National Health Service Trust Research Ethics Committee"* in the methods section.

Referee #2:

Subtypes of NF-κB are essential transcription factors to regulate inflammatory responses to pathogens. Here Bartlett et al demonstrate the functional role of NF-κB p65 in rhinovirus (RV) infection. Inhibition of NF-κB p65 using siRNA-mediated knockdown did not alter the RV-induced IFNs but reduced the production of inflammatory cytokine and chemokines such as CCL5, CXCL8 and CXCL5. Consistent with this, IκB DN expression decreased IL-6 induction but not IFN-β and λ1. Utilizing p65 heterozygous mice, whose expression level of p65 is much reduced compared to wild type, they clearly showed the same tendency in in-vivo RV infection. Neutrophilic inflammation and production of chemokines were impaired in p65 heterozygous mice, while IFN induction and virus loads were unaffected. In summary, they provide a new insight into RV infection and suggest a therapeutic potential of NF-κB p65 for the treatment of RV-induced airway diseases.

Minor comment:

They mention about p65 deficient mice in page 10: "Consistent with HBEC in vitro data p65 deficient mice did not show reduced levels of type I or type III IFN following RV infection in vivo...."

It would be nice to include these data or provide a proper reference for this.

Response: We thank the reviewer for their support and interest. The effects of p65 deficiency on RV induced IFNs *in vivo* is included in Fig 4.

Referee #3:

In this manuscript Bartlett et al examined the role of NF-kB p65 in type I interferon production after Rhinovirus infection. The authors discovered that RV infection induces p65 activation but p65 is not required for IFN-b or IFN-lambda expression. However, they found that p65 is required for airway inflammation. This is a potentially interesting observation since inhibitors of p65 would not affect

the antiviral response but could diminish airway inflammation. The role of NF-kB in IFN-b activation is debated for the last years and many conflicting reports appear in the literature. The current picture says that p65 is required for the early antiviral response and before the entry of IRF3 in the picture. This issue has not been addressed in the current manuscript experimentally by carrying out detailed time-courses. Furthermore some of the biochemical data are not of sufficient quality to fully justify the authors' conclusions. For example, the quality of Figure 1A should be improved and labelled appropriately to indicate nuclear activation and the EMSA should be shown in Fig. 2A together with the appropriate NF-kB subunit identification experiments (antibody super shift experiments) to substantiate the involvement of p65 in the NF-kB complexes formed after virus infection. In other words, it could be that p65 together with other Rel proteins are co-induced and could replace each other. Finally, the paper jumps from human to mouse cells and then back to human and then back to mouse models to substantiate the authors' conclusions. Although these are important steps to validate the clinical role of components it appears that generate a lot of confusion for molecular-mechanistic studies since it could imply species-specific variability. In summary, a more streamlined version of the manuscript could be more informative and interesting for the general audience.

<u>Response:</u> We thank the reviewer for their support and interest. Regarding the first point, we agree we have not looked at early time points in human cells *in vitro*, however we feel we have investigated adequate time courses *in vivo*. *In vivo* we investigated 8, 24, 48 and 96h and did not find reduced IFN responses in $p65^{+/-}$ mice compared to controls. Furthermore, there was no difference in ISG or RV replication at any time point, indirect evidence that IFN responses are comparable to wildtypes in this model. We would argue, if there were diminished IFN responses at any time point (including early time points) we would observe increased RV, or reduced ISGs later; however did not observe this at all. In support, $IFNAR1^{-/-}$ mice are a good example of what diminished IFN responses do look like in RV infection; these give higher virus loads and diminished ISG responses. Also of note, Wang et al (Wang, JImmunol 2010) did observe increased NDV replication at later points in $p65^{-/-}$ cells, due to diminished early IFN. However, in our model there is no evidence to support diminished early IFN responses, and we feel our experimental design and data is therefore thorough enough to conclude that the IFN response is similar between $p65^{+/+}$ and $p65^{+/-}$ mice.

We have added arrows to Figure 1A to help discriminate cells with nuclear p65 staining.

We have addressed the additional point made by the reviewer by performing p65-DNA binding assays during RV infection *in vivo*, using Bl/6 129 mice, thereby justifying the claim that p65 is activated by RV *in vivo*. The p65-DNA binding studies at 8h are shown in supplementary data Fig S4 and S5. We have elected to leave the gel shift picture in Fig 1, as over all this data supports the fact that NF-kB is activated by RV *in vivo*. We would further add that we agree that other NF-kB family members may substitute for p65 in IFN induction; however we have not attempted to address this issue in this study and in our view is a separate question. What is absolutely compelling by our studies is the differential requirement of p65 for IFN versus the inflammatory responses, therefore making p65 a target for intervention.

We would again disagree with the reviewer regarding the combining of mouse and human data; we argue the message of each figure is clear and addresses different aspects of p65 biology in RV infection. The combination of mouse and human data only strengthens the manuscript and furthermore the mouse and human data support each other, the fact that p65 is dispensable for IFN in human cells *in vitro* is also true *in vivo,* and this is the important message we would like to convey to the reader. We argue the mixing of human and mouse data provides a clear and important message and would be appealing to a wide audience.

2nd Editorial Decision 17 September 2012

Thank you for the submission of your revised manuscript "Defining critical roles for NF- B p65 and type I interferon in innate immunity to rhinovirus" to EMBO Molecular Medicine. We have now received the reports from the reviewer who was asked to re-review your manuscript.

You will be glad to see that the reviewer is now globally supportive and we can proceed with official acceptance of your manuscript pending the minor change detailed below:

- The description of all reported data that includes statistical testing must state the name of the statistical test used to generate error bars and P values, the number (n) of independent experiments underlying each data point (not replicate measures of one sample), and the actual P value for each test (not merely 'significant' or 'P < 0.05').

Please submit your revised manuscript latest within two weeks. I look forward to seeing a revised form of your manuscript as soon as possible.

Yours sincerely,

Editor
EMBO Molecular Medicine

***** Reviewer's comments *****

Referee #1:

Overall, this is an important and well controlled study. It exhibits significant translational potential in terms of future drug development for viral exacerbations of asthma.

2nd Revision - Authors' Response 19 September 2012

We would like to thank you for the acceptance of our manuscript. Regarding an explanation of the statistical analysis, we have followed the journals instructions as best as we could, however as we have used parametric statistics for most of the analysis (including one-way and two-way ANOVA with Bonferroni's multiple comparison test) our statistics package does not report actual p-values but rather gives P<0.05, P<0.01 etc. I do not think that this poses a problem however as this is used by other articles published in the journal (I refer to Koltida, 2010, and O'Donoghue, current issue, EMBO Mol Med 2012). We have amended each figure legend such that the statistical test is named, and p-value explanations follow after each figure panel and that the data is expressed as mean±standard error of mean. Our figure legends also include number of replicates and independent experiments used. Our statistics section in the methods also does state the statistics employed, software package used numbers of replicates and numbers of independent experiments for both in vivo and in vitro experiments. I have updated the manuscript file on the online submission system, and all changes are in red. I hope these changes are acceptable to the journal.
