# Supplementary material for: Defining critical roles for NF-κB p65 and type I interferon in innate immunity to rhinovirus
Source: EMBO Mol Med. 2012 Nov 14;4(12):1244–60. doi: 10.1002/emmm.201201650 (PMC3531601; doi:10.1002/emmm.201201650)
Supplement: Supplementary file 2 [file emmm0004-1244-SD2.pdf]

Supplementary Information

Supplementary Information 1

Figure S1: NF-κB p65 specific siRNA reduced p65 mRNA and protein in HBECs. 2

Figure S2: IRF3 specific siRNA reduced IRF3 mRNA and protein respectively in HBECs. 2

Figure S3: UV-inactivated RV does not induce cell recruitment to BAL. 3

Figure S4: NF-κB *p65*$^{+/-}$ mice have reduced p65 protein compared to NF-κB *p65*$^{+/+}$ mice. 4

Figure S6: Analysis of CD3+ and NK cells in *IFNAR1*$^{-/-}$ and wt mice by flow cytometery. 6

Table SI: Effect of IRF3, NF-κB p65, siRNA or dsDNA transfection on IFN-β, IFN-λ1 and IFN-λ2/3 mRNA expression in HBECs 7

Table SII: Effect of NF-κB p65 siRNA or dsDNA transfection on CCL5, CXCL5, and CXCL8 mRNA expression in HBEC. 8

Table SIII: Effect of NF-κB p65 siRNA, control siRNA or dsDNA transfection on Rel family member mRNA expression in HBECs. 9

Table SIV: Effect of IRF3 siRNA, control siRNA or dsDNA transfection on IRF family member mRNA expression in HBECs. 10

Table SV. Primer and Probe sequences for use in Realtime RT-PCR. 11

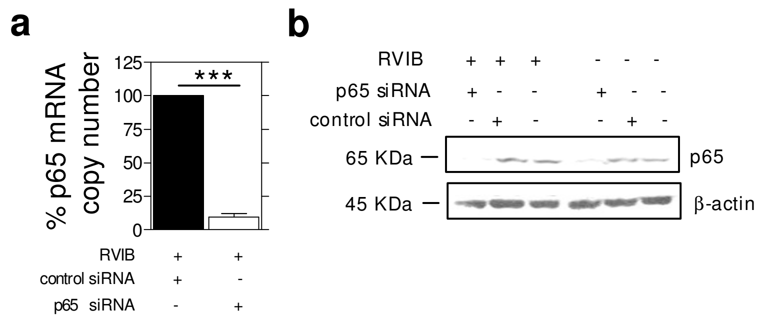

Figure S1: NF-κB p65 specific siRNA reduced p65 mRNA and protein in HBECs. (a) Transfection with siRNA specific to NF-κB p65 but not control siRNA reduced p65 mRNA 24h post infection with RV1B. (b) NF-κB specific siRNA reduced p65 protein 24h post infection with RV1B or treatment with medium. ****P*<0.001 as indicated.

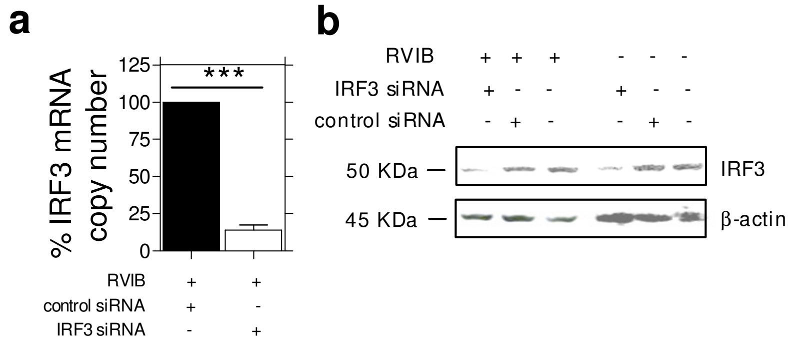

Figure S2: IRF3 specific siRNA reduced IRF3 mRNA and protein respectively in HBECs. (a) Transfection with siRNA specific to IRF3 but not control siRNA reduced IRF3 mRNA 24h post infection with RV1B. (b) IRF3 specific siRNA reduced IRF3 protein 24h post infection with RV1B or treatment with medium. ****P*<0.001 as indicated.

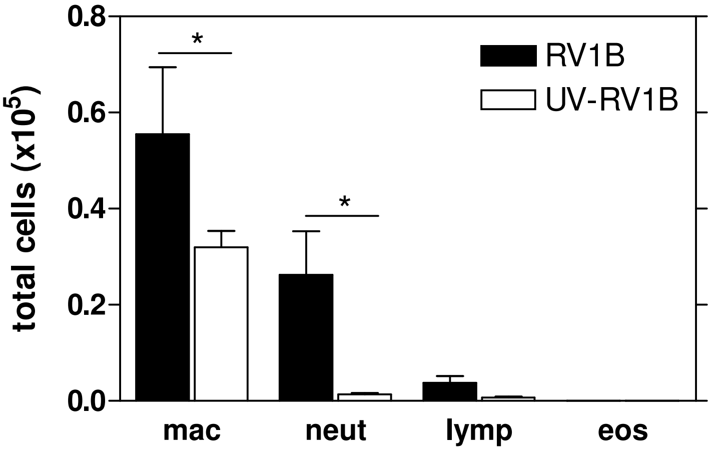

Figure S3: UV-inactivated RV does not induce cell recruitment to BAL. Wildtype C57Bl/6 mice were infected with preparations of purified RV1B, or UV-inactivated purified RV (UV-RV1B) intranasally. At 24h post infection, BAL was harvested, cell number was enumerated and cell type analysed by cytospin and H&E staining. Immune cells were identified on the basis of H&E staining and scored as either macrophages, neutrophils, lymphocytes or eosinophils. Experiments consisted of 2-4 mice per group, performed 4 times for a total of n=10 mice. *P<0.05 as indicated.

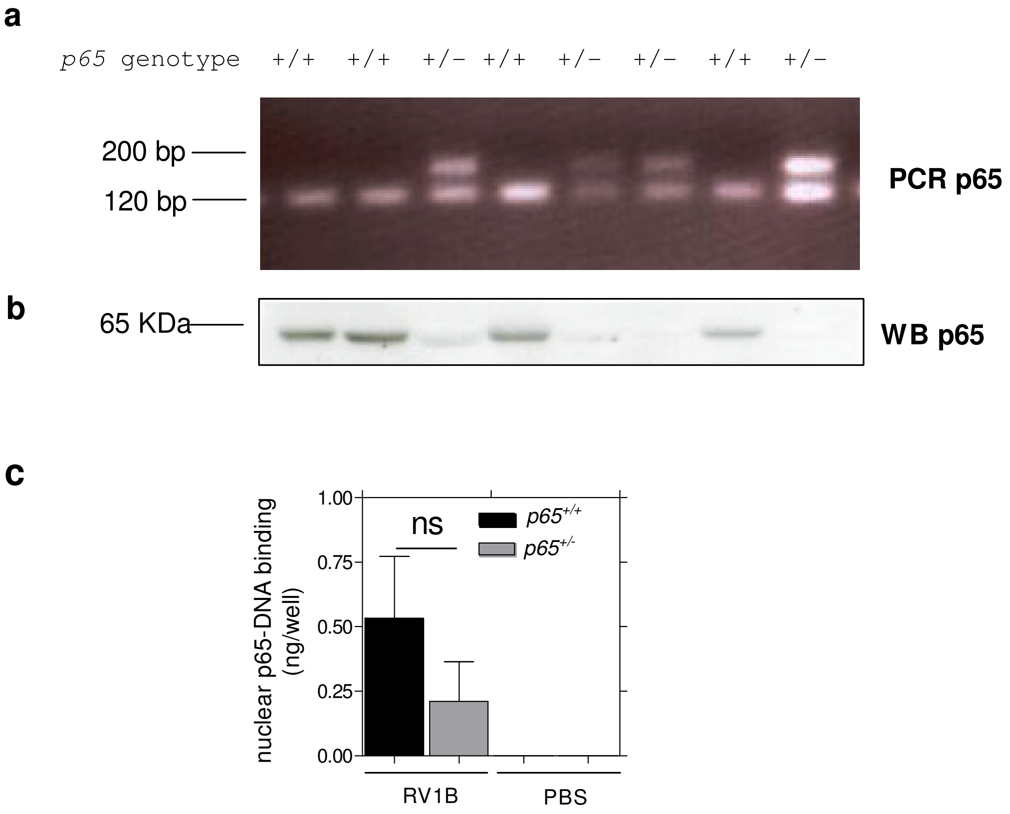

Figure S4: NF-κB ***p65*** $^{+/-}$ **mice have reduced p65 protein compared to NF-κB** ***p65*** $^{+/+}$ **mice.** (a) Mice were genotyped using specific PCR for mouse p65 DNA. *P65* $^{+/+}$ exhibit one band of approximately 120bp, while herterozygote *p65* $^{+/-}$ give two distinct bands, including the 120bp band derived from the wildtype gene and a 200bp band derived from the transgene. (b) Cells were isolated from BAL of *p65* $^{+/+}$ and *p65* $^{+/-}$ mice and presence of p65 protein analysed by western blot. NF-κB *p65* $^{+/+}$ had more p65 protein when compared with *p65* $^{+/-}$ mice. (c) *p65* $^{+/+}$ and *p65* $^{+/-}$ mice were infected with RV1B or PBS via the intranasal route and at 8h post infection, the lung was sampled for p65 activation. RV1B infected *p65* $^{+/+}$ mice had more activated p65 as assessed by p65-DNA binding than *p65* $^{+/-}$ or mice treated with PBS. NS= not significant.

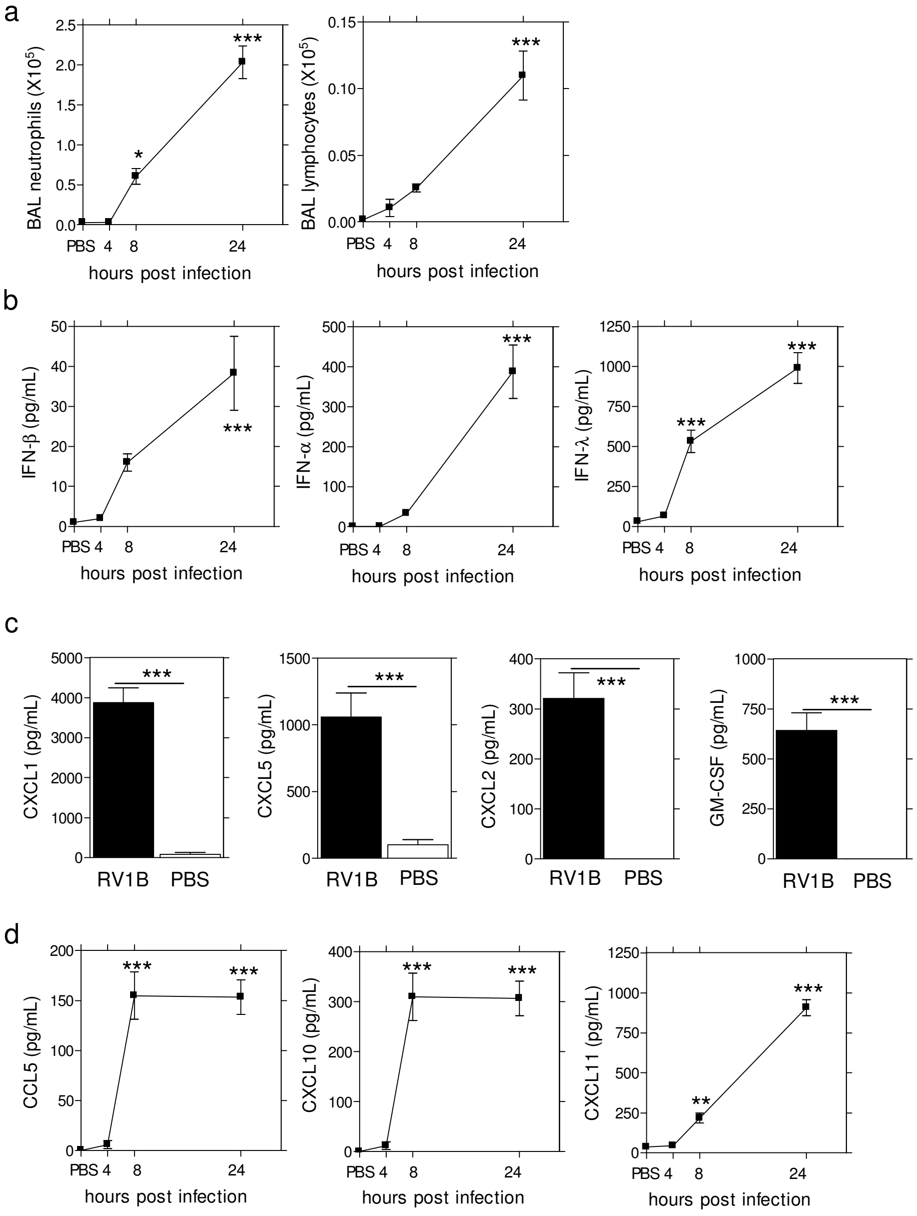

Figure S5: RV induced neutrophil and lymphocyte recruitment, IFNs, pro-inflammatory cytokine and chemokine production in wildtype Bl/6 129 mice. Bl/6 129 mice were infected with RV1B or PBS, intranasally. At 4, 8 and 24h post infection, BAL was harvested for cell number (a) IFNs (b) and cytokines (c,d). Experiments consisted of 5 mice per group, performed once. **P*<0.05,***P*<0.01, ****P*<0.001 as indicated or versus PBS control.

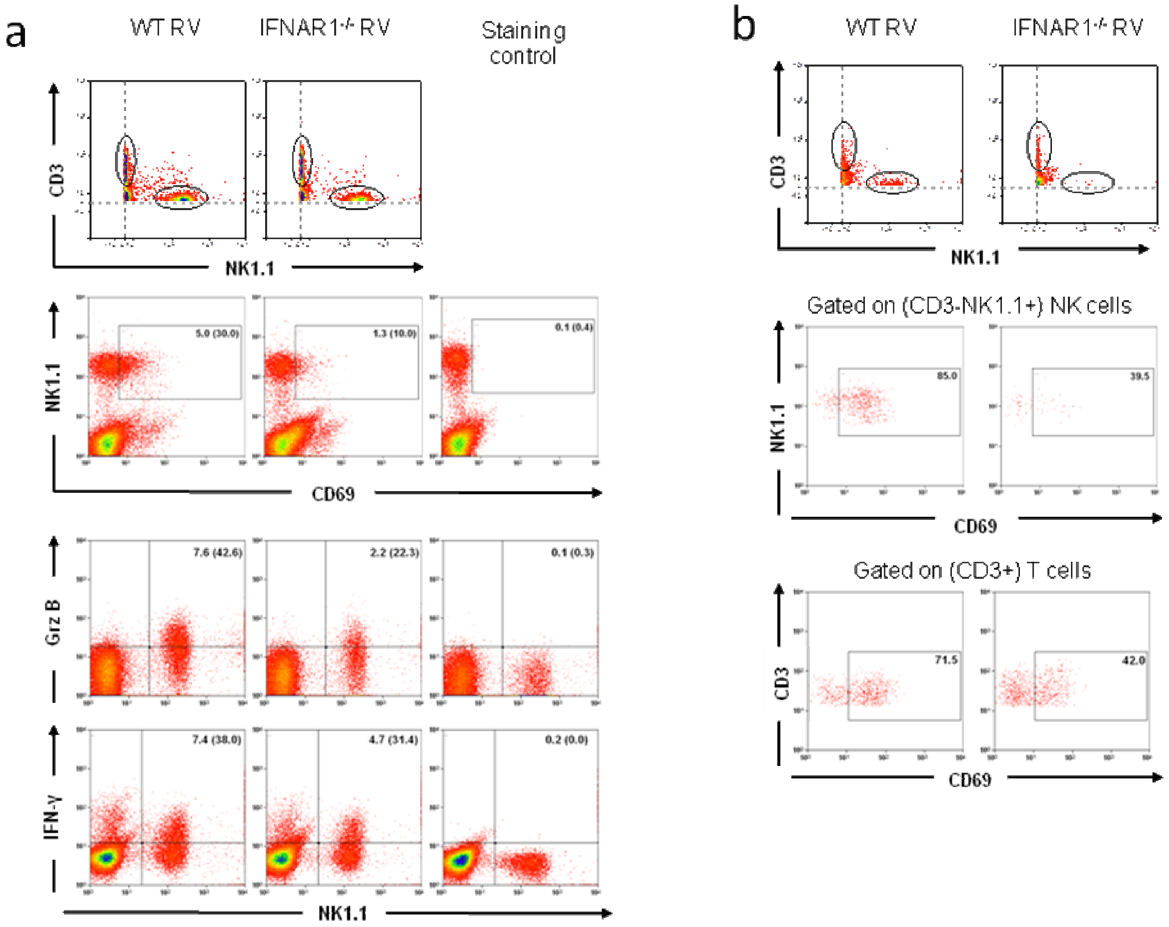

Figure S6: Analysis of CD3+ and NK cells in *IFNAR1$^{-/-}$* and wt mice by flow cytometery. Representative gating for lung and BAL flow cytometry staining in RV infected wild type and *IFNAR1$^{-/-}$* mice. Lung tissue (a) or BAL cells (b) were isolated on day 3 after infection and either surface stained directly for CD3, NK1.1 and CD69, or stimulated for 3h with PMA and Ionomycin and also stained for intracellular Grz B and IFN-γ (as described in methods). Numbers on plots represent the % of cells falling within the indicated gate, and in parenthesis the percentage of NK cells specifically. All plots were gated on viable, forward scatter/side scatter determined lymphocytes.

Table SI: Effect of IRF3, NF-κB p65, siRNA or dsDNA transfection on IFN-β, IFN-λ1 and IFN-λ2/3 mRNA expression in HBECs. HBECs were transiently transfected with IRF3, p65 siRNA or a dsDNA oligo as a transfection control. 24h post-transfection, medium was replaced and cells cultured in fresh medium for 24h. Total cell RNA was harvested for analysis at 48h post-transfection and IFN-β, IFN-λ1 and IFN-λ2/3 measured by TaqMan RT-PCR. IRF3, p65 and dsDNA transfection did not significantly induce IFN-β, IFN-λ1 or IFN-λ2/3 mRNA expression compared to un-transfected control cells (all P>0.05). Data are the mean mRNA copy number per $2x10^5$ cells ± SEM of 4 separate experiments performed in three different HBEC donours. Comparisons are presented as dsDNA versus untransfected cells (^{NS}), and IRF1, IRF3, or NF-κB p65 siRNA versus dsDNA/untransfected cells respectively ($^{NS/NS}$).

	**IFN-β**	**IFN-λ1**	**IFN-λ2/3**
**Un-transfected**			
Mean copies ± SEM	$2.0x10^4 \pm 7.6x10^3$	$6.7x10^2 \pm 4.8x10^2$	$1.2x10^3 \pm 6.2x10^2$
**dsDNA**†			
Mean copies ± SEM	$3.1x10^4 \pm 1.3x10^{4\ NS}$	$2.2x10^3 \pm 1.5x10^{3\ NS}$	$1.9x10^3 \pm 1.0x10^{3\ NS}$
**IRF3 siRNA**			
Mean copies ± SEM	$1.1x10^4 \pm 3.2x10^{4\ NS/NS}$	$2.2x10^3 \pm 1.6x10^{3\ NS/NS}$	$2.1x10^2 \pm 1.0x10^{2\ NS/NS}$
**NF-κB p65 siRNA**			
Mean copies ± SEM	$3.5x10^4 \pm 1.1x10^{4\ NS/NS}$	$6.1x10^2 \pm 3.7x10^{2\ NS/NS}$	$6.0x10^2 \pm 2.5x10^{2\ NS/NS}$

†dsDNA is an oligo designed on the AP-1 binding site within the human IL-8 promoter.

Table SII: Effect of NF-κB p65 siRNA or dsDNA transfection on CCL5, CXCL5, and CXCL8 mRNA expression in HBEC. HBEC were transiently transfected with NF-κB p65 siRNA or a dsDNA oligo as a transfection control. 24h post-transfection, medium was replaced and cells cultured for a further 24h. Total cell RNA was harvested for analysis at 48h post-transfection and pro-inflammatory cytokines measured by TaqMan RT-PCR. NF-κB p65 siRNA or dsDNA transfection did not significantly induce CCL5, CXCL5, or CXCL8 mRNA expression compared to un-transfected cells (all P>0.05). Data are the mean mRNA copy number per $2x10^5$ cells ± SEM of 4 separate experiments performed in three different HBEC donours. Comparisons are shown as dsDNA versus untransfected cells (^{NS}), and NF-κB p65 targeting siRNA versus dsDNA/untransfected cells respectively ($^{NS/NS}$).

	**CCL5**	**CXCL5**	**CXCL8**
**Un-transfected**			
Mean copies	$5.0x10^5$	$4.8x10^5$	$3.3x10^6$
± SEM	± $1.6x10^5$	± $1.2x10^5$	± $1.2x10^6$
**dsDNA**[†]			
Mean copies	$1.0x10^{6\ NS}$	$7.8x10^{5\ NS}$	$3.9x10^{6\ NS}$
± SEM	± $4.0x10^5$	± $2.1x10^5$	± $1.0x10^6$
**NF-κB p65 siRNA**			
Mean copies	$1.1x10^{5\ NS/NS}$	$2.9x10^5$ NS/NS	$9.3x10^{5\ NS/NS}$
± SEM	± $4.9x10^4$	± $1.0x10^5$	± $3.0x10^5$

[†]dsDNA is an oligo designed on the AP-1 binding site within the human IL-8 promoter.

Table SIII: Effect of NF-κB p65 siRNA, control siRNA or dsDNA transfection on Rel family member mRNA expression in HBECs. HBECs were transiently transfected with p65 siRNA control siRNA a dsDNA oligo as a transfection control or left untransfected. At 24h post-transfection, medium was replaced and cells cultured in fresh medium for 24h. Total cell RNA was harvested for analysis at 48h post-transfection and p52, RelB, c-Rel and p50 mRNA measured by TaqMan RT-PCR. p65 siRNA did not significantly affect any Rel family member mRNA expression compared to untransfected control cells or control siRNA transfected cells (all *P*>0.05). Data are the mean fold induction ± SEM versus medium of 4 separate experiments performed in three different HBEC donours. Comparisons are presented as NF-κB p65 siRNA versus untransfected cells$^{(NS)}$ or p65 siRNA versus untransfected and control siRNA$^{(NS/NS)}$.

	p52	RelB	c-Rel	p50
**Un-transfected**				
Mean fold induction	1.00	1.00	1.00	1.00
**dsDNA†**				
Mean fold induction ± SEM	1.22 ± 0.29$^{(NS/NS)}$	1.57 ± 0.48$^{(NS/NS)}$	0.92 ± 0.07$^{(NS/NS)}$	1.02 ± 0.31$^{(NS/NS)}$
**Control siRNA**				
Mean fold induction ± SEM	1.27 ± 0.85$^{(NS/NS)}$	1.36 ± 0.38$^{(NS/NS)}$	0.87 ± 0.37$^{(NS/NS)}$	0.74 ± 0.56$^{(NS/NS)}$
**NF-κB p65 siRNA**				
Mean fold induction ± SEM	1.39 ± 0.63$^{(NS/NS)}$	1.51 ±0.63$^{(NS/NS)}$	1.98 ± 0.50$^{(NS/NS)}$	1.02 ± 0.34$^{(NS/NS)}$

†dsDNA is an oligo designed on the AP-1 binding site within the human IL-8 promoter.

Table SIV: Effect of IRF3 siRNA, control siRNA or dsDNA transfection on IRF family member mRNA expression in HBECs. HBECs were transiently transfected with IRF3 siRNA, control siRNA a dsDNA oligo as a transfection control or left untransfected. At 24h post-transfection, medium was replaced and cells cultured in fresh medium for 24h. Total cell RNA was harvested for analysis at 48h post-transfection and IRF1, IRF5, IRF7 and IRF9 mRNA measured by TaqMan RT-PCR. IRF3 siRNA did not significantly affect any IRF family member mRNA expression compared to untransfected control cells or control siRNA transfected cells (all *P*>0.05). Data are the mean fold induction ± SEM versus medium of 4 separate experiments performed in three different HBEC donours. Comparisons are presented as IRF3 siRNA versus untransfected cells$^{(NS)}$ or IRF3 siRNA versus untransfected and control siRNA$^{(NS/NS)}$.

	IRF1	IRF5	IRF7	IRF9
**Un-transfected**				
Mean fold induction	1.00	1.00	1.00	1.00
**dsDNA†**				
Mean fold induction ± SEM	1.05 ± 0.09$^{(NS/NS)}$	1.06 ± 0.12$^{(NS/NS)}$	1.53 ± 0.36$^{(NS/NS)}$	1.75 ± 0.32$^{(NS/NS)}$
**Control siRNA**				
Mean fold induction ± SEM	1.70 ± 0.72$^{(NS/NS)}$	1.17 ± 0.19$^{(NS/NS)}$	1.67 ± 0.41 $^{(NS/NS)}$	1.54 ± 0.60$^{(NS/NS)}$
**IRF3 siRNA**				
Mean fold induction ± SEM	12.35 ± 10.64 $^{(NS/NS)}$	2.37 ± 0.94$^{(NS/NS)}$	2.09 ± 0.75 $^{(NS/NS)}$	4.89 ± 2.7$^{(NS/NS)}$

†dsDNA is an oligo designed on the AP-1 binding site within the human IL-8 promoter.

Table SV. Primer and Probe sequences for use in Realtime RT-PCR.

Gene	Primer/ Probe	Sequence (5'-3')
**Human IFN-β NM_002176**	Forward	CGCCGCATTGACCATCTA
	Reverse	TTAGCCAGGAGGTTCTCAACAATAGTCTCA
	Probe	FAM-TCAGACAAGATTCATCTAGCACTGGCTGGA-TAMRA
**Human IFN-λ1 NM_172140**	Forward	GGACGCCTTGGAAGAGTCACT
	Reverse	AGAAGCCTCAGGTCCCAATTC
	Probe	FAM-AGTTGCAGCTCTCCTGTCTTCCCCG-TAMRA
**Human IFN-λ2/3 NM_172139**	Forward	CTGCCACATAGCCCAGTTCA
	Reverse	AGAAGCGACTCTTCTAAGGCATCTT
	Probe	FAM-TCTCCACAGGAGCTGCAGGCCTTTA-TAMRA
**Human CXCL8 NM_000584**	Forward	CTGGCCGTGGCTCTCTTG
	Reverse	CCTTGGCAAAACTGCACCTT
	Probe	FAM-CAGCCTTCCTGATTTCTGCAGCTCTGTGT-TAMRA
**Human CXCL5 NM_002994**	Forward	AGAGCTGCGTTGCGTTTGT
	Reverse	TGGCGAACACTTGCAGATTACT
	Probe	FAM-ACAGACCACGCAAGGAGTTCATCCCA-TAMRA
**Human CCL5 NM_002985**	Forward	GCATCTGCCTCCCCATATTC
	Reverse	CAGTGGGCGGGCAATG
	Probe	FAM-TCGGACACCACACCCTGCTGCT-TAMRA
**Human p65 NM_021975**	Forward	CGAACTGTTCCCCCTCATCTT
	Reverse	CTTGGGCTGCTCAATGATCTC
	Probe	FAM-CCGGCAGAGCCAGCCCAGG-TAMRA
**Human p50 NM_003998.3**	Forward	CCATACCTTCAAATATTAGAGCAACCT
	Reverse	TGGGATGGGCCTTCACATA
	Probe	FAM-CAGAGAGGATTTCGTTTCCG-TAMRA
**Human Rel B NM_006509**	Forward	GCGGATTTGCCGAATTAACA
	Reverse	TGTCCTCTTTCTGCACCTTGTC
	Probe	FAM-GAGCAAGTAGAGCTCCTCGCCA-TAMRA

**Human p52** **NM_001077494.2**	Forward<br>Reverse<br>Probe	CCCATCTGCGCCGTTTCT<br>ACATGCAGGACACCCAGGTT<br>FAM-TTAAATTGGGCAGTCATGTCCTTGG-TAMRA
**Human c-Rel** **NM_002908.2**	Forward<br>Reverse<br>Probe	TCCCTGATGAACATGGTAATTTGA<br>AGTATTTGGAGCACGGTTGTCA<br>FAM-ACTGCTCTTCCTCCTGTTGTCTCGAACCC-TAMRA
**Human IRF3** **NM_001571.5**	Forward<br>Reverse<br>Probe	GCTCGTGATGGTCAAGGTTGT<br>CCTACCCGGGCCATTTCT<br>FAM-CCAAGGCCCTGAGGCACGTGG-TAMRA
**Human IRF1** **NM_002198.2**	Forward<br>Reverse<br>Probe	CTCCAGCACTGTCGCCATG<br>GCACAACTTCCACTGGGATGT<br>FAM-TGTCAGCAGCACTCTCCCCGACTG-TAMRA
**Human IRF5** **NM_032643**	Forward<br>Reverse<br>Probe	CCCCCAGAGCTGGTTGTTAA<br>CTGGAGTGTGCAGAGATGACACA<br>FAM-AGCCTGGCACCTACCCGCTCTCA-TAMRA
**Human IRF7** **NM_001572.3**	Forward<br>Reverse<br>Probe	TCCCCACGCTATACCATCTACCT<br>ACAGCCAGGGTTCCAGCTT<br>FAM-CTTCGGGCAGGACCTGTCAGCTG-TAMRA
**Human IRF9** **NM_006084.4**	Forward<br>Reverse<br>Probe	GCCCTACAAGGTGTATCAGTTGCT<br>TCGCTTTGATGGTACTTTCTGAGT<br>FAM-CCACCAGGAATCGTCTCTGGCCA-TAMRA
**RV** **In house**	Forward<br>Reverse<br>Probe	GTGAAGAGCCSCRTGTGCT<br>GCTSCAGGGTTAAGGTTAGCC<br>FAM-TGAGTCCTCCGGCCCCTGAATG-TAMRA
**Mouse IFN-β** **NM_010510**	Forward<br>Reverse<br>Probe	CCATCATGAACAGGTGGAT<br>GAGAGGGCTGTGGTGGAGAA<br>FAM-CTCCACGCTGCGTTCCTGCTGTG-TAMRA
**Mouse IL-28** **NM_0010246**	Forward	AAAGGATTGCCACATTGCTC

**73**	Reverse<br>Probe	TCAAGCAGCCTCTTCTCGAT<br>FAM-TCCCCAAAAGAGCTGCAGGC-TAMRA
**Mouse COX-2**<br>**NM_011198**	Forward<br>Reverse<br>Probe	TGGCGCTCAGCCATACAG<br>TCCGGGTACAATCGCACTTAT<br>FAM-TGTTCCCACCCATGTCAAAACCGAG-TAMRA
**Mouse OAS1A**<br>**NM_145211**	Forward<br>Reverse<br>Probe	TCCTGGGTCATGTTAATACTTCCA<br>CCCCAGGGAGGTACATTCCT<br>FAM-CAAGCCTGATCCCAGAATCTATGCC-TAMRA
**Mouse Viperin *(CIG5)***<br>**AF236064**	Forward<br>Reverse<br>Probe	CGAAGACATGAATGAACACATCAA<br>AATTAGGAGGCACTGGAAAACCT<br>FAM-CCAGCGCACAGGGCTCAGGG-TAMRA
**Mouse PKR**<br>**NM_011163**	Forward<br>Reverse<br>Probe	CGGAACATCCTCTAGCGTTGTC<br>GGGAAACACCATTACTTGTCATAGAC<br>FAM-AGCTGCTGGAAAAGCCACTGA-TAMRA
**18S rRNA**<br>**M10098**	Forward<br>Reverse<br>Probe	CGCCGCTAGAGGTGAAATTCT<br>CATTCTTGGCAAATGCTTTCG<br>FAM-ACCGGCGCAAGACGGACCAGA-TAMRA
